# 2-Azahetaryl-2-(oxoindolin-2-ylidene)acetonitriles as Colorimetric Probes for Zn: Synthesis and Optical Properties

**DOI:** 10.1021/acsomega.2c04747

**Published:** 2022-11-16

**Authors:** Vladyslav V. Shcherban, Olena O. Kuleshova, Tetiana Ye. Keda, Olga V. Khilya, Emmanuel Gras, Yulian M. Volovenko

**Affiliations:** † Faculty of Chemistry, Taras Shevchenko National University of Kyiv, Lva Tolstoho Street 12, Kyiv01033, Ukraine; ‡ Laboratoire de Chimie de Coordination, Centre National de la Recherche Scientifique, UPR 8241, Université Fed́eŕale Toulouse Midi-Pyreńeés, 205, Route de Narbonne, ToulouseF-31077, France

## Abstract

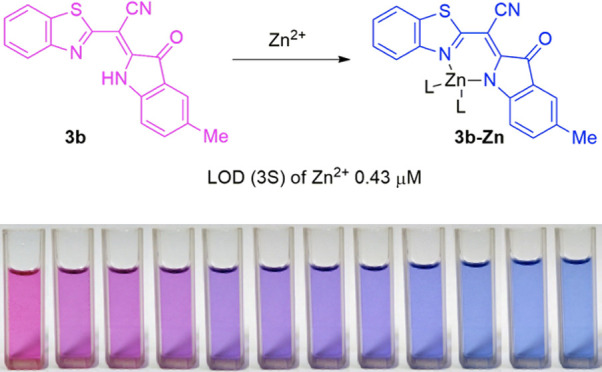

A new one-pot approach for the synthesis of the Zn^2+^-sensitive probes 2-azahetaryl-2-(oxoindolin-2-ylidene)acetonitriles **3a–c** and **4** is described. The method includes the *in situ* formation of imidoylchloride and its further condensation with azahetarylacetonitrile **1**. The structure of the obtained compounds is studied using ^1^H nuclear magnetic resonance (NMR), ^13^C NMR, infrared (IR), high-resolution mass spectrometry (HRMS), and UV–Vis spectroscopy techniques. Two model ligands both exhibiting the highest extinction coefficient and the best solubility in a Tris buffer pH 7.2/dimethyl sulfoxide (DMSO) solution, namely 5-methyl-benzothiazole derivative **3b** and benzoxazole derivative **4**, are thoroughly studied as colorimetric probes for Zn^2+^. The probe **3b** has the highest sensitivity to Zn^2+^, showing a limit of ion detection (LOD) calculated by the 3S criterion of 0.43 μM and selectivity upon masking Cu^2+^ ions with Na_2_S_2_O_3_. The composition of the complexes in the solution was determined by the limited logarithm method. The stability constant (lg *K*) values of **3b-Zn** of 10.27 ± 0.02 and **4-Zn** of 12.5 ± 0.2 indicate the formation of complexes of average stability.

## 1 Introduction

Zinc is widely recognized as a “gatekeeper of the immune system”^1^ that protects the human body against diseases and possesses antioxidant, anti-inflammatory, and antiviral activities,^2^ including against Sars-Cov2.^3−7^ To assess the individual or population zinc status, various biomarkers are recommended, in particular, concentration of zinc in plasma or serum.^8−10^

**Figure 1:**
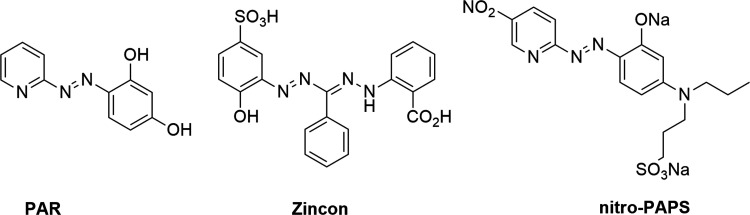
Structure of well-known chromophore probes for zinc.

From a research perspective, the recommended methods for the determination of Zn^2+^ in biofluids are atomic absorption spectroscopy and inductively coupled plasma mass spectrometry.^11,12^ However, these methods are too expensive to be involved in routine clinical analysis. Fluorescence analysis is also a widely used technique;^13^ however, UV–VIS spectrophotometry (SP) remains the state-of-the-art technique for biochemical and clinical research due to its accuracy, sensitivity, and simplicity. Despite the large number of chromophore probes for zinc,^14^ a limited number of them are used for serum and plasma SP analysis. Well-known ones in the range are PAR^15^ and Zincon,^16^ but they either do not have contrasting color transitions or sufficient selectivity or require toxic masking agents such as cyanide ions, for example, in serum analysis with nitro-PAPS^17^ (Figure 1).

**Figure 2:**
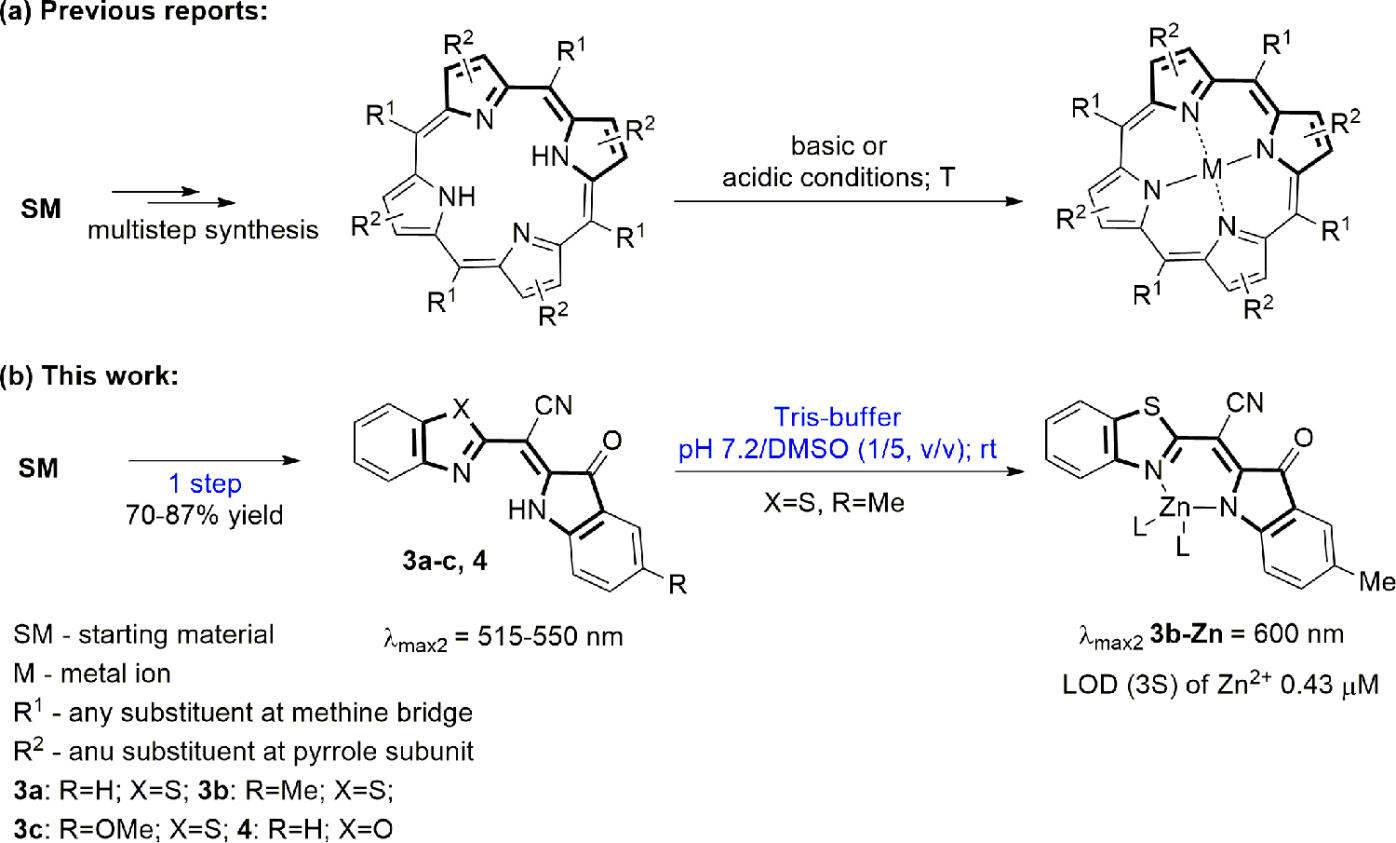
(a, b) Porphyrinoids and their simplified analogues as metal probes.

It is known that porphyrinoids because of their large conjugated system have a strong absorption peak in the visible region.^18^ This feature correlates with their wide use as a sensitive chromogenic reagent for the detection of metal traces.^18−22^ However, their coordination to metals often requires elevated temperature, auxiliaries, or pH far from biologically relevant conditions,^18,19^ probably because of the rigidity of the cavity and the required deprotonations (Figure 2a). Moreover, fast functionalization of porphyrins suffers from significant synthetic limitations; thus, switching their coordination and optical properties is troublesome. Multistep synthesis of the probe is indeed usually required.^23^

Here, we report simplified nonmacrocyclic analogues of porphyrins, 2-azahetaryl-2-(oxoindolin-2-ylidene)acetonitriles **3a–c** and **4** that are easily available by a one-pot three-component reaction. Featuring the dipyrrin-like skeleton, they exhibit the potential for metal chelation. The representative probe **3b** can chelate Zn^2+^ at room temperature (hereafter rt) in Tris buffer pH 7.2/DMSO (1/5, v/v) with a LOD equal to 0.43 μM and selectivity upon masking Cu^2+^ ions (Figure 2b).

## 2 Results and Discussion

### 2.1 Synthesis

To modulate the spectral characteristics of colorimetric probes, we aimed at simplifying the structure of the well-known porphyrins. Thus, we envisaged that 2-azahetaryl-2-(oxoindolin-2-ylidene)acetonitriles **3a–c** and **4** might be competitive with porphyrins in terms of light absorption, while they could be synthesized from commercially available isatines **2** and *N*-hetarylacetonitriles **1** easily accessed synthetically from the readily available starting materials. Moreover, kinetics of the coordination of Zn^2+^ might be enhanced because of the decreased steric hindrance at the coordination site.

It is known that the reaction of isatines with nucleophiles is directed at position 3 of isatin. To direct the reaction at position 2, a preactivation can be achieved by refluxing isatin and PCl_5_ in benzene. The resulting imidoylchloride is so far, requiring isolation and dissolution in more polar solvents to perform the condensation reaction. This two-step approach decreases the yield of the product due to the poor stability of imidoylchloride, promoting the formation of byproducts.^24−26^

**Scheme 1:**
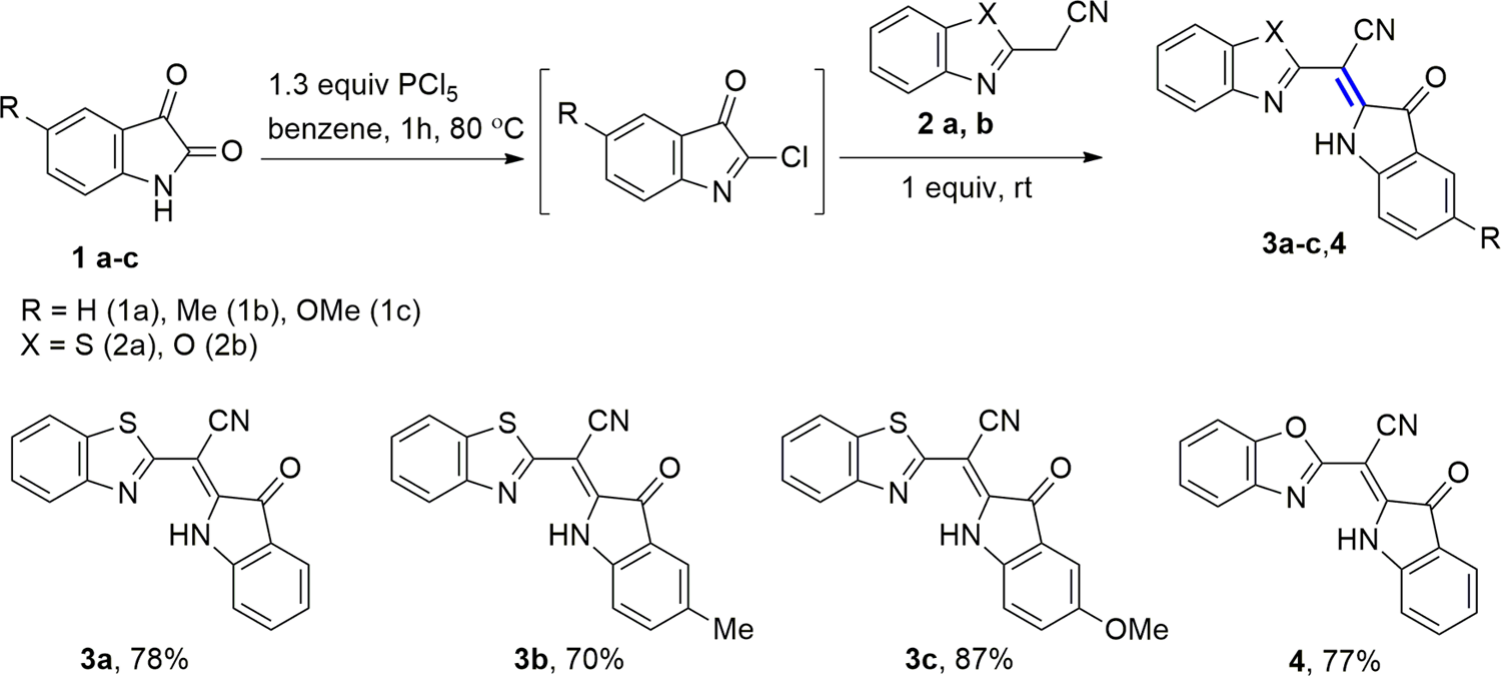
Synthesis of Dyes **3a–c** and **4**

To overcome this limitation, we have reported a one-pot method consisting first of*in situ* formation of imidoylchloride followed by its condensation with hetarylacetonitrile **1** (Scheme 1).^27^ Similar three-component reactions were applied for the synthesis of 2-azahetarylenaminonitriles as reported earlier by our group.^28^

**Figure 3:**
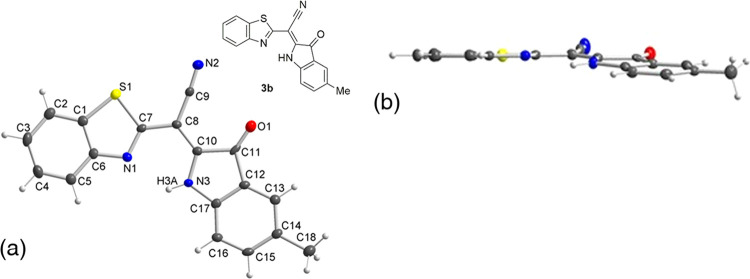
Molecular structure of **3b** according to X-ray diffraction with the atom numbering used in the crystallographic analysis. (a) Planar projection and (b) orthogonal projection.

The limiting reaction step of the process is the generation of imidoylchloride, yet the reaction is over within an hour. The yield of the products ranges from 70 to 87%, and their purification is achieved by simple filtration through a plug of silica gel using dichloromethane as an eluent. In the ^1^H NMR spectra of **3a–c** and **4**, the characteristic broad peak of the proton of the amino group is significantly deshielded (δ = 11.4–12.0 ppm). Most likely, the reason for this downfield shift is an intramolecular hydrogen bond that forms between the nitrogen of the azaheterocycle and the hydrogen of the indolinone backbone. Single crystals of **3b** are grown by a gas diffusion method from CHCl_3_/hexane; XRD allows the full assessment of the molecular structure (Figure 3).

According to the XRD of **3b**, the estimated length of the hydrogen bond N3H3A···N1 is 2.15 Å and the angle N3–H3A···N1 is equal to 123.3°. The dihedral angle between heterocycle planes is equal to 8.8°, which indicates an effective charge delocalization in the molecule, promoting the shift of the NH in ^1^H NMR to the deshielded side.

A complete assignment of the peaks at ^1^H and ^13^C NMR was performed using 2D NMR techniques (COSY, HMBC, and HMQC).

The method showed a wide scope allowing to generate a library of ligands to modulate the donating properties of the R substituent and the nature and electronic properties of the attached heterocycle. Thus, a library of compounds has been accessed based on either benzothiazole (**3**) or benzoxazole (**4**) from a reaction with unsubstituted isatin (**a**), isatin featuring a σ-donor such as a methyl group (**b)**, or a π-donor such as a methoxy group (**c**).

### 2.2 Photophysical Properties

**Figure 4:**
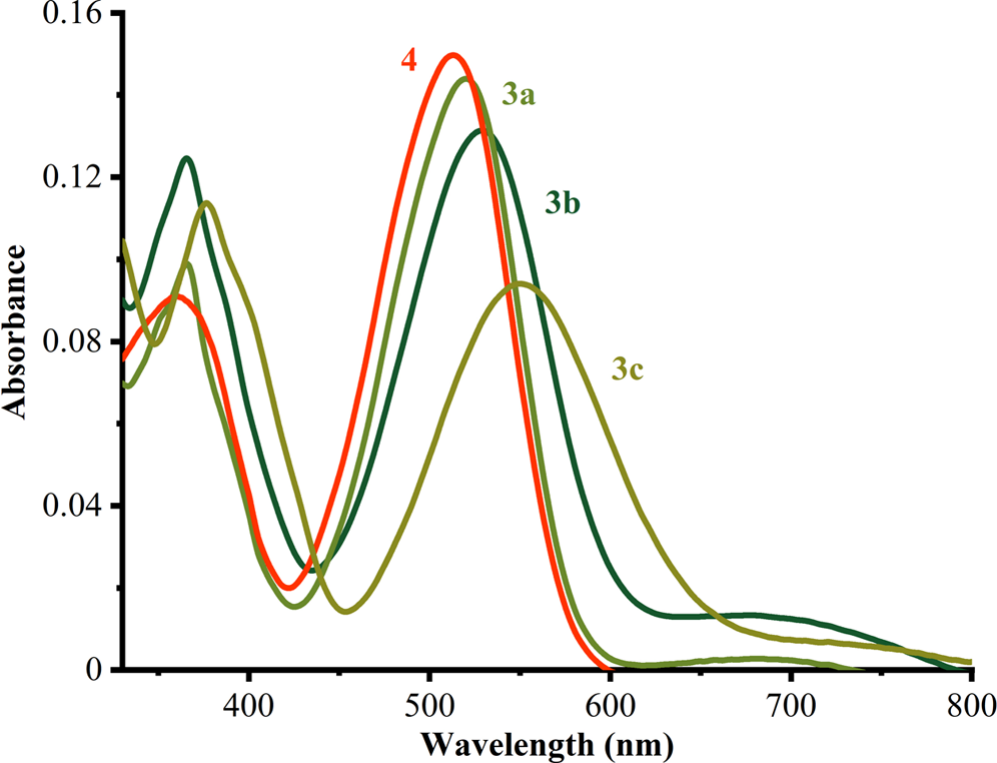
Absorption spectra of dyes **3a–c** and **4** in DMSO solution. *C* = 10 μmol·L^–1^ and *l* = 1.00 cm.

2-Azahetaryl-2-(5-R-3-oxoindoline-2-ylidene)acetonitriles are characterized by the rich color ranging from red to purple. The nonsubstituted isatin compounds **3a** and **4** in DMSO featured absorption spectra with a long wave band at 425–600 nm and the maximum centered around 520 and 515 nm, respectively (Figure 4 and Table 1). Methyl- and methoxy-substituted compounds **3b** and **3c** in DMSO featured absorption spectra with the maximum centered around 530 and 550 nm, respectively, and featuring less intensive peaks at 650–750 nm. The increased bathochromic shift of the substituted dyes compared to nonsubstituted ones may be rationalized by the presence of electron density donors at the oxoindolinone core consequently shrinking of the HOMO–LUMO gap.

**Table 1:** Spectrophotometric Characteristics of Dyes **3a–c** and **4** in DMSO Solution

dye	λ_max1_, nm	ε_λmax1_, mol^–1^ L cm^–1^, 10^4^	λ_max2_, nm	ε_λmax2_, mol^–1^ L cm^–1^, 10^4^
**3a**	363	1.10 ± 0.11	520	1.48 ± 0.05
**3b**	367	1.37 ± 0.07	530	1.38 ± 0.05
**3c**	376	1.26 ± 0.05	550	1.03 ± 0.05
**4**	357	1.09 ± 0.14	515	1.66 ± 0.10

### 2.3 Photostability Test

To assess the stability of dyes in DMSO solutions, UV–Vis absorption spectra were recorded after 24 h, 72 h, 96 h, and 6 months of exposure to indirect light. The absorbance of the solutions remains unchanged, which indicates a high photostability of the dye solutions (see Figure S14).

### 2.4 UV–Vis Spectra in H_2_O/DMSO Solutions

Water solubility of 2-azahetaryl-2-(5-R-3-oxoindolin-2-ylidene)acetonitriles affects seriously their spectroscopic and chemical properties. The influence of water on the absorption spectra of the dyes **3a**, **3b**, and **4** in DMSO and the values of their molar absorption coefficient (ε) were studied (see Supporting Information Section 3). The molar absorption coefficient change was calculated as Δε_max_, % = (ε_max DMSO_ – ε_max H_2_O/DMSO_) × 100/ε_max DMSO_, where ε_max DMSO_ and ε_max H_2_O/DMSO_ are the molar absorption coefficient of dyes at λ_max_ in DMSO and H_2_O/DMSO mixtures with different solvent ratios (v/v), respectively. Apparently, dyes **3b** and **4** were found to be the most hydrophilic compounds in H_2_O/DMSO (1/(1.5 to 5), v/v) mixtures. The decrease of the molar absorption coefficient was lower than 10% (Δε_max_ ≤ 10%), while a decrease of the absorbance value up to 50% was observed for dye **3a** (see Table S2). Based on this observation, dyes **3b** and **4** were chosen for further investigations.

**Figure 5:**
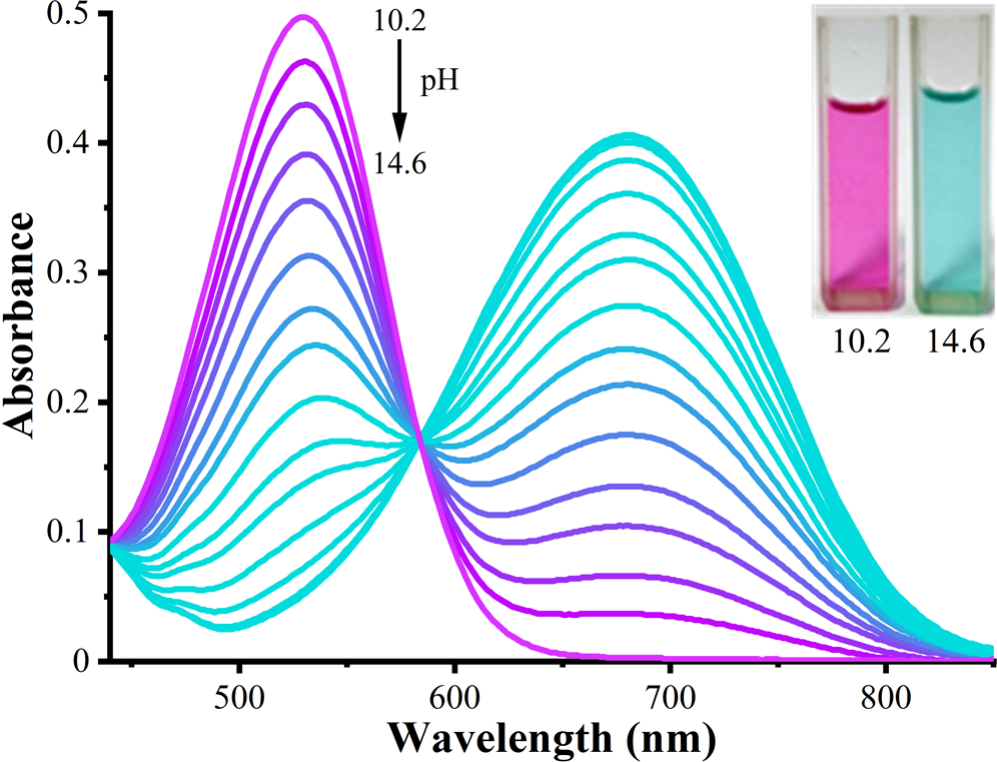
Absorption spectra of 40 μmol·L^–1^**3b** in H_2_O/DMSO (1/5, v/v) upon addition of different amounts of NaOH. Photographs of neutral (left) and anionic (right) forms of dye in solution with appropriate pH are shown in the inset. *l* = 1.00 cm.

**Figure 6:**
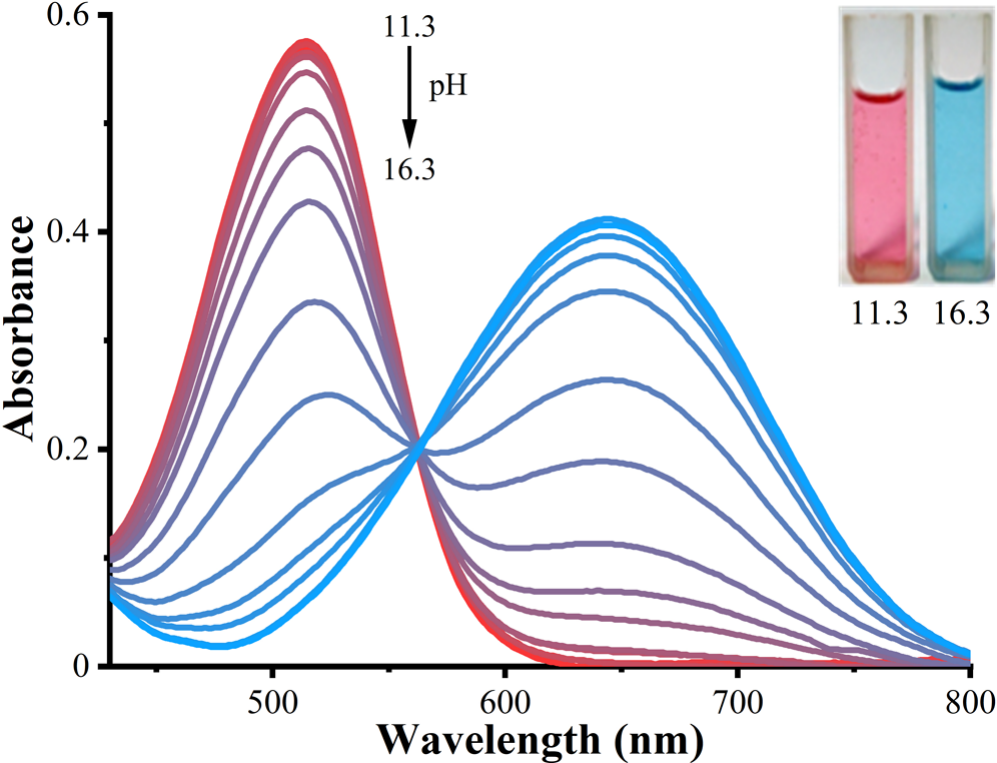
Absorption spectra of 40 μmol.L^–1^**4** in H_2_O/DMSO (1/3, v/v) upon addition of different amounts of NaOH. Photographs of neutral (left) and anionic (right) forms of dye in solution with appropriate pH are shown in the inset. *l* = 1.00 cm.

The deprotonation of molecules **3b** and **4** at the NH site upon titration with alkaline solution is demonstrated in Figures 5 and 6. The isosbestic points located at 584 nm for **3b** and 562 nm for **4** indicate the coexistence of two forms (neutral and anionic) of dyes in alkaline solutions. The bathochromic shifts (Δλ) of the absorption bands of neutral forms (λ_max_ = 530 nm (**3b**) and λ_max_ = 515 nm (**4**)) to anionic forms are equal to 150 nm for **3b** and 130 nm for **4**, which correspond to the drastic color change from purple to turquoise visible by the naked eye. These transitions were shown to be reversible. The bathochromic shift occurring under basic conditions may be rationalized by the formation of an anion, characterized by the facilitated oscillation of the π-electrons compared to the neutral form. Therefore, less energy is required to excite the electrons, which causes a long-wavelength π → π* electronic transition (see Scheme S1).

The apparent ionization constant (p*K*_a_) values of molecules **3b** and **4** in H_2_O/DMSO mixtures have been obtained with spectrophotometric titrations according to eq 1 (Section 4.3) (Tables 2, S4, and S5).

**Table 2:** Apparent Ionization Constants of the Dyes **3b** and **4** in H_2_O/DMSO Solutions

dye	H_2_O/DMSO(v/v)	p*K*_a_
**3b**	1/5	13.7 ± 0.1
**4**	1/3	15.2 ± 0.2

### 2.5 Interaction with Zn^2+^

**Figure 7:**
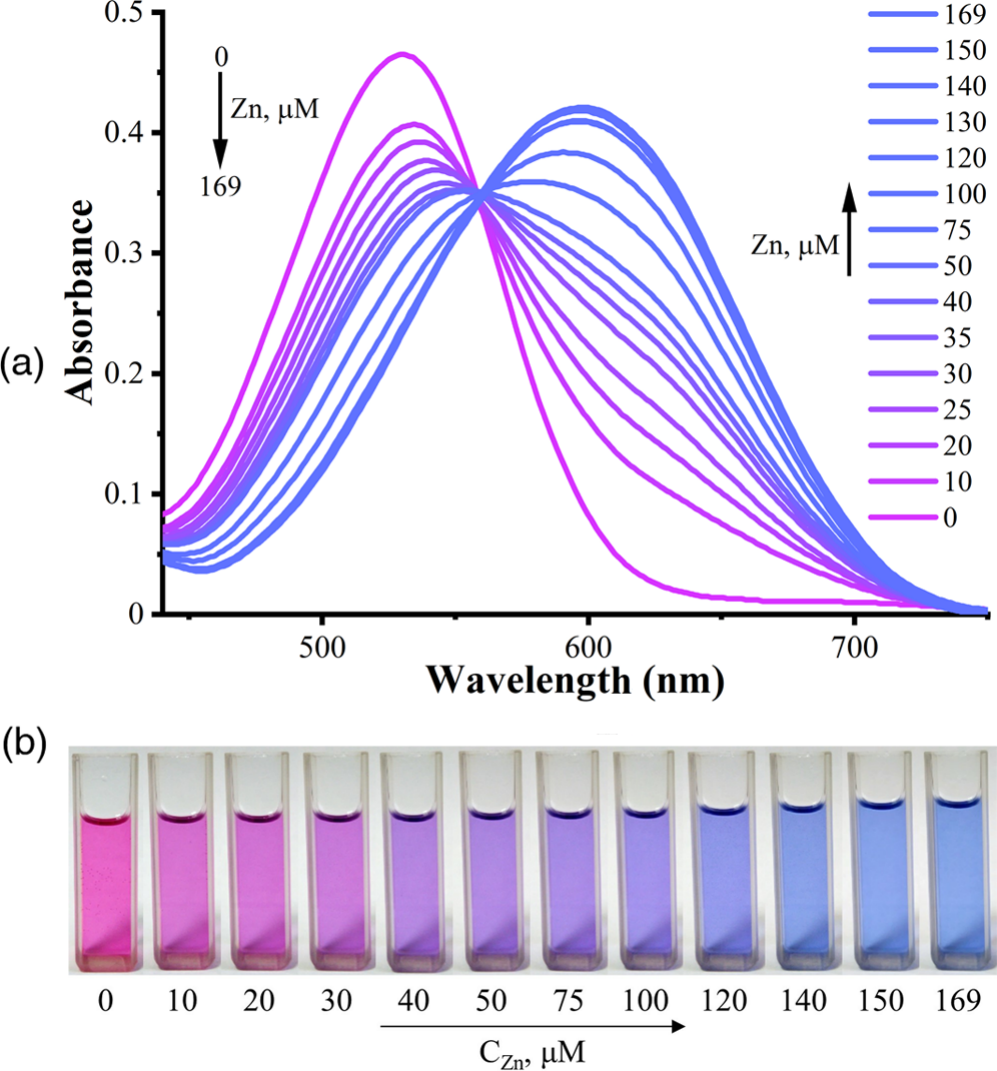
Absorption spectra of dye **3b** without and in the presence of different amounts of Zn^2+^, *C*_**3b**_ = 38 μmol·L^–1^, Tris buffer (pH 7.2)/DMSO (1/5, v/v) (a), photographs of dye **3b** in the presence of different amounts of Zn^2+^ (b); concentration of Zn^2+^ is shown in the insets.

**Figure 8:**
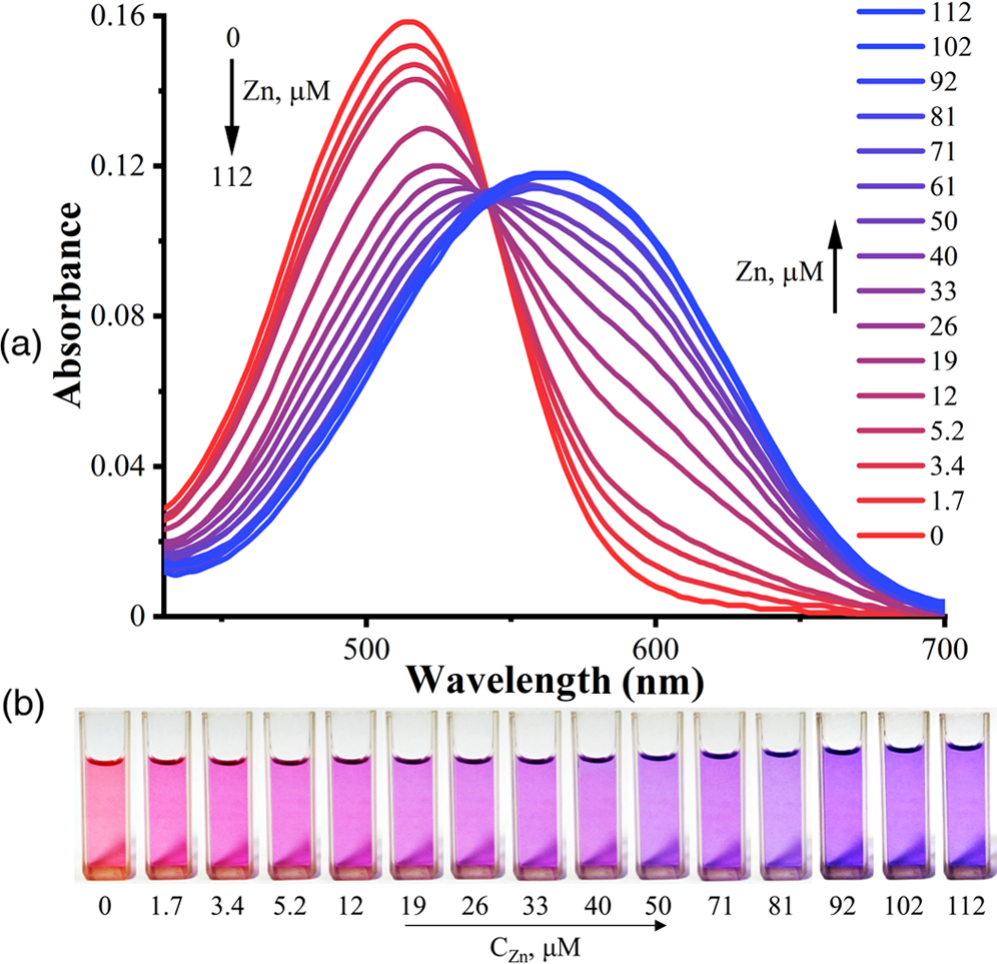
Absorption spectra of dye **4** without and in the presence of different amounts of Zn^2+^, *C*_**4**_ = 9.9 μmol·L^–1^, Tris buffer (pH 7.2)/DMSO (1/3, v/v) (a); photographs of dye **4** in the presence of different amounts of Zn^2+^ (b); concentration of Zn^2+^ is shown in the insets.

It was envisioned that dyes **3b** and **4** can chelate Zn^2+^ due to the presence of the dipyrrin-like skeleton.^29^ Indeed, upon addition of Zn^2+^ to the solution of **3b** and **4**, the hyperchromic effect occurs at 600 and 565 nm, respectively. Meanwhile, the color changes from purple (**3b**) and red (**4**) to blue, which corresponds to the bathochromic shift of λ_max_ in the UV–vis spectra (Figures 7 and 8). Such changes may probably indicate the formation of complexes. The most convenient environment for the interaction of the ligand and Zn^2+^ was chosen based on two factors: the largest achievable light absorption of the complex at the appropriate wavelength (to ensure the highest detection sensitivity) and the lowest possible DMSO content. Keeping this in mind and considering the data presented in Figures S17b and S18 and Table S3, the ratios of Tris buffer (pH 7.2) and DMSO of 1/5 (**3b**) and 1/3 (**4**) were the best.

**Figure 9:**
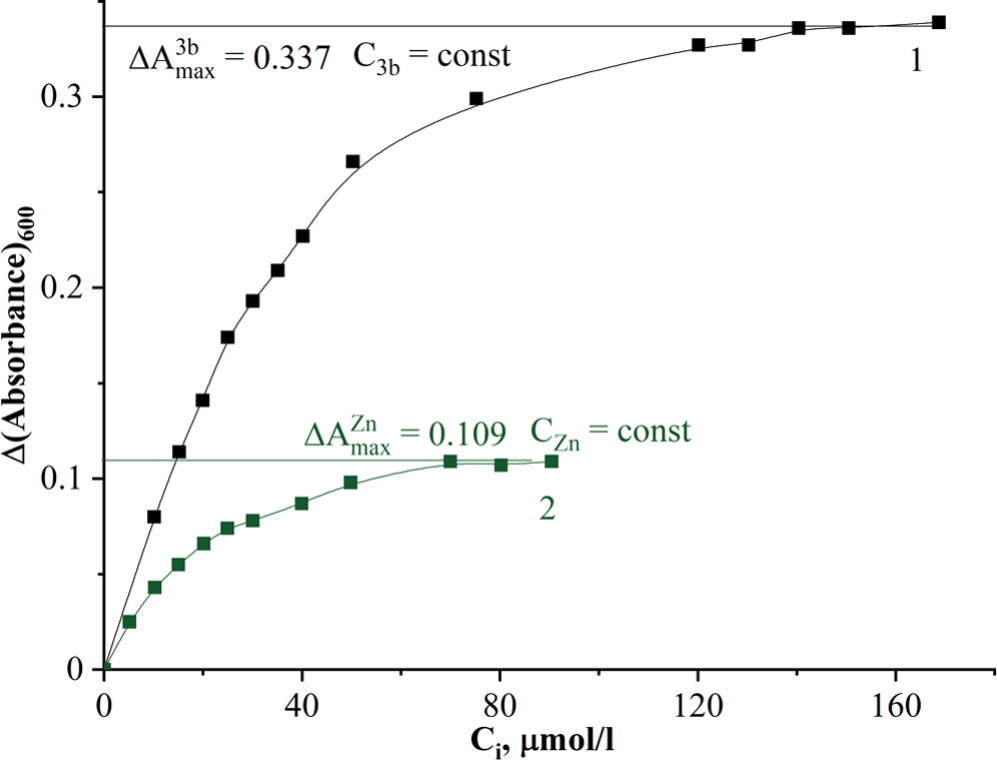
Saturation curves of the complex formation between Zn^2+^ and probe **3b** in Tris buffer (pH 7.2)/DMSO (1/5, v/v). *C*(**3b**) = 38 μmol·L^–1^ (1); *C*(Zn^2+^) = 10 μmol·L^–1^ (2).

**Figure 10:**
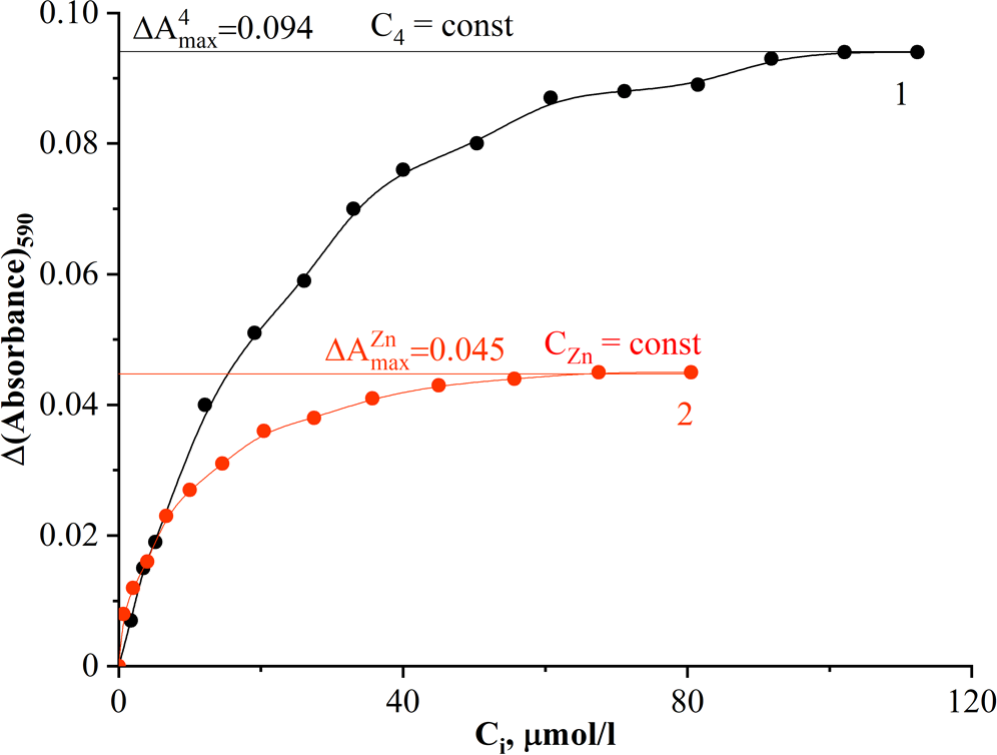
Saturation curves of the complex formation between Zn^2+^ and probe **4** in Tris buffer (pH 7.2)/DMSO (1/3, v/v). *C*(**4**) = 10 μmol·L^–1^ (1) and *C*(Zn^2+^) = 5 μmol·L^–1^ (2).

The classic Bent and French method^30^ was applied for spectral data processing. Considering the shape of the saturation curves in Figures 9 and 10, it can be assumed that a medium stability complex was formed in the solutions. The molar composition of complexes (Zn*_m_*R*_n_*) was established as described in Section 4.4. The calculations gave the values of *n*/*m* equal to 1 for both complexes of probes **3b** and **4** with Zn^2+^, which confirmed the formation of ZnR complexes in solutions. Under these conditions, the neutral forms of probes appeared to be predominant in the solutions (see Figure S19).

The stability constants of the complexes **3b-Zn** and **4-Zn** have been calculated by processing the data from the saturation curves (Figures 9 and 10) using eq 2 (see Supporting Information Section 5). The values of the affinity constants were found to be equal lg *K*^f^ = 10.27 ± 0.02 and lg *K*^f^ = 12.5 ± 0.2 for probes **3b** and **4**, respectively.

### 2.6 Spectrophotometric Determination of Zn^2+^

Hence, the absorbance of probes **3b** and **4** at the maximum of the complexes after treatment with the Zn^2+^ solutions was also found to be dependent on their concentration. The dependence of the absorption maxima and absorbance at the wavelength ranges with a minimum spectrum overlap is consistent with the Beer–Lambert law in the wide Zn^2+^ concentration ranges (Table 3).

**Table 3:** Performance Characteristics of Zn^2+^ Spectrophotometric Determination (Δ*A*_λ_ = *A*_λi_ – *A*_λ0_, *n* = 6, *P* = 0.95)

dye	regression equation	RSD^2^	linear range, μmol·L^–1^	LOD (3S), μmol·L^–1^
**3b**	Δ*A*_640_ = (0.003 ± 0.001) + (0.0103 ± 0.0001)·*C*_Zn_, μmol·L^–1^	0.999	1.0–20.0	0.43
	Δ*A*_600_ = (0.005 ± 0.002) + (0.0111 ± 0.0002)·*C*_Zn_, μmol·L^–1^	0.998		0.54
**4**	Δ*A*_610_ = (0.0013 ± 0.0009) + (0.0028 ± 0.0002)·*C*_Zn_, μmol·L^–1^	0.992	1.7–12.1	0.96
	Δ*A*_590_ = (0.002 ± 0.001) + (0.0032 ± 0.0002)·*C*_Zn_, μmol·L^–1^	0.990		0.94

The dye **3b** exhibits better characteristics due to the wider linearity of the Zn^2+^ concentration range and higher sensitivity. The selectivity of dyes **3b** toward Zn^2+^ was appraised by titration of the probe with various metal ions under the optimal conditions of zinc complex formation (see Supporting Information Section 6). It was shown that heavy metal ions such as Fe^3+^, Fe^2+^, Ni^2+^, and Cd^2+^ did not affect the absorption of dye **3b** (see Figure S22). As expected, only Co^2+^ and Cu^2+^ caused significant change in the colors of the solutions. However, the change of the color in the presence of Co^2+^ is a very slow process and the absorption spectrum change could be observed after 30 min of solution treatment with Co^2+^ (see Figure S23). Due to this fact, the presence of an equal amount of Co^2+^ does not interfere with the Zn^2+^ determination during 20 min after the probe treatment. It was found that Cu^2+^ ions could be masked using 1.0 mmol·L^–1^ Na_2_S_2_O_3_ solution (see Figure S24).

To prove the reliability of the technique, the probe **3b** was applied for the detection of Zn^2+^ in the serum model solution (stripped of protein) (see Supporting Information Section 7). The model solution of two times diluted pretreated blood serum was prepared as recommended in ref (31) using standard solutions of Zn^2+^, Cu^2+^, Fe^2+^, Na^+^, K^+^, Ca^2+^, Mg^2+^, and Tris buffer (pH = 7.2). The resulting samples were analyzed spectrophotometrically. For this purpose, 2.5 mL of 40 μmol·L^–1^ of probe **3b** solution in DMSO was mixed with 0.5 mL of model solution in Tris buffer (pH = 7.2) in the presence of Na_2_S_2_O_3_, and the absorbance of the solutions was measured. The recovery value does not exceed 105% for 5.0 and 10.0 μmol·L^–1^ spiked Zn^2+^ in the sample. The data obtained suggest that the proposed technique could be used for Zn^2+^ determination in real samples. Full method validation using real samples will be the aim of our future work.

A comparison of probe **3b** with other reported colorimetric probes for Zn^2+^ in complex matrices has shown that the closest analogue of probe **3b** in terms of sensitivity, metal ion selectivity, and contrasting color change accompanying the complexation is Zincon (see Table S9). Moreover, probe **3b** demonstrates much better stability in solutions than Zincon.^16^ Finally, a high DMSO content in the sample solution does not interfere with the determination of the Zn total amount in the pretreated biosamples using probe **3b**.

## 3 Conclusions

In summary, we have developed a one-pot approach for the synthesis of 2-azahetaryl-2-(oxoindolin-2-ylidene)acetonitriles via *in situ* formation of imidoylchloride, and we have found that these dyes are sensitive probes for Zn^2+^ ions when Cu^2+^ ions are masked with Na_2_S_2_O_3._ The best analytical response is achieved with benzothiazole derivative **3b** with a LOD of 0.43 μM in a Tris buffer pH 7.2/DMSO solution. The contrasting color change accompanied with the complex formation and the stability of the dye and its complex with zinc facilitate the highly sensitive and selective determination of Zn^2+^ ions using UV–Vis spectrophotometry. Therefore, probe **3b** is promising for detecting and monitoring Zn^2+^ ions in biological fluids, such as serum.

## 4 Experimental Section^32^

### 4.1 Materials and Methods

All chemicals and solvents were analytical grade and used without further purification. Isatin **2a**, 5-methylisatin **2b**, and 5-methoxyisatin **2c** were provided by Enamine Ltd. 2-(Benzo[*d*]thiazol-2-yl)acetonitrile **1a** was synthesized by a known method.^33^ 2-(Benzo[*d*]thiazol-2-yl)acetonitrile **1b** was synthesized as described in Section 4.2.

Preparative chromatography was performed manually with silica gel (63–200 μm).

^1^H NMR and ^13^C{^1^H} NMR spectra were recorded on a Bruker Avance 300 (300 and 75 MHz, respectively) or Bruker Avance III 400 (400 and 101 MHz, respectively) spectrometer and were referenced to the residual proton resonances of the DMSO-*d*_6_: 2.50 for ^1^H and 40.0 ppm for ^13^C. The degree of carbon atom substitution was determined by NMR spectra acquired according to the DEPT-135 or JMOD methods. Two-dimensional (2D) (COSY, HMQC, HMBC) spectra were recorded on a Bruker Avance III 400 spectrometer and Bruker Avance 500. All spectra were recorded at ambient temperature (298 K). Coupling constants (*J*) were reported in Hz, and chemical shift (δ) were reported in ppm. The multiplicity of signals is indicated using the following abbreviations: s = singlet, bs = broad singlet, d = doublet, t = triplet, q = quartet, quint = quintet, dd = doublet of doublets, ddd = doublet of doublets of doublets, dt = doublet of triplets, m = multiplet, and br = broad.

IR spectra were recorded on a FT-IR spectrometer Perkin Elmer in KBr plates.

High-resolution mass spectra (HRMS) were recorded on a GCT Premier spectrometer upon electron spray ionization (ESI) or chemical ionization (DCI, CH_4_).

Melting points were determined on a Boetius micro-hot stage apparatus with a VEB Analytik 1399RNMK 05 observation lens.

X-ray crystallography was performed on a single-crystal diffractometer Agilent Gemini. A gas diffusion method was implemented for growing single crystals.

Absorption spectra were recorded on a SHIMADZU UV-2401 PC spectrophotometer. pH measurements were performed with a STARTER 3100 bench pH meter (OHAUS).

### 4.2 Synthesis

#### 4.2.1 2-(Benzo[*d*]oxazol-2-yl)acetonitrile

A solution of 2-aminophenol (4.36 g, 40 mmol) and 2-cyanoacetimidate hydrochloride^34^ (6.36 g, 44 mmol) in anhydrous CH_2_Cl_2_ (110 mL) was heated to reflux over a period of 10 h (TLC elution system: CHCl_3_/CH_3_OH (9:1)). The reaction mixture was then cooled, diluted with CH_2_Cl_2_ (110 mL), and washed with 10% NaOH solution until discoloration of the aqueous layer. The organic layer was dried over MgSO_4_ and concentrated under reduced pressure to yield the desired compound. A total of 4.43 g of the product (28 mmol, 70 % yield) was obtained as a beige solid. ^**1**^**H NMR (400 MHz, chloroform-*****d*****)** δ 4.13 (s, 2H), 7.37–7.45 (m, 2H), 7.55–7.60 (m, 1H), 7.74–7.79 (m, 1H). The compound was used in the next step without further purification.

#### 4.2.2 General Procedure for the Synthesis of 2-Hetaryl-2-(5-R-3-oxoindolin-2-ylidene)acetonitriles **3a–c** and **4**

To a strongly stirred suspension of 5-R-isatine (1 mmol) in hot benzene (10–15 mL), phosphorus pentachloride (1.3 mmol) was added. The resulting mixture was refluxed for 1 h. Then, a solution of hetarylacetonitrile (1 mmol) in benzene was added dropwise and a characteristic color change occurred. The mixture was allowed to cool to room temperature upon stirring. The precipitate was then filtered and washed with minimum amounts of ethanol and water and again with ethanol. The dry precipitate was purified by filtration through a plug of silica gel (eluent: CH_2_Cl_2_).

#### 4.2.3 (*Z*)-2-(Benzo[*d*]thiazol-2-yl)-2-(3-oxoindolin-2-ylidene)acetonitrile **3a**

The product (236 mg, 0.78 mmol, 78% yield) was obtained as a dark-red powder (mp >300 °C) from 147 mg (1 mmol) of isatin **1a** and 174 mg (1 mmol) of 2-(benzo[*d*]thiazol-2-yl)acetonitrile **2a. IR (KBr):** 3408, 2205, 1712, 1589. ^**1**^**H NMR (400 MHz, DMSO-*****d***_**6**_**)** δ 7.16 (td, *J* = 7.5, 0.8 Hz, 1H, 5-H_Is_), 7.50 – 7.56 (m, 2H, 5-H_Bt_, 7-H_Is_), 7.66 (ddd, *J* = 8.2, 7.8, 1.1 Hz, 1H, 6-H_Bt_), 7.66–7.71 (m, 1H, 6-H_Is_), 7.73 (d, *J* = 7.6 Hz, 1H, 4-H_Is_), 8.20 (d, *J* = 8.1, 1.0, 0.6 Hz, 1H, 7-H), 8.21 (d, *J* = 8.0, 1.3, 0.6 Hz, 1H, 4-H), 11.55 (s, 1H, NH). ^**13**^**C{**^**1**^**H} NMR (101 MHz, DMSO-*****d***_**6**_**)** δ 80.2 (C), 114.5 (CH), 116.9 (C), 119.9 (C), 123.0 (CH), 123.2 (CH), 123.6 (CH), 125.7 (CH), 126.4 (CH), 127.6 (CH), 134.3 (C), 138.2 (CH), 143.7 (C), 151.9 (C), 154.0 (C), 163.4 (C), 185.1 (C). **HRMS** (ESI) calcd for C_17_H_10_N_3_OS (M + H^+^) 304.0545, found 304.0545.

#### 4.2.4 (*Z*)-2-(Benzo[*d*]thiazol-2-yl)-2-(5-methyl-3-oxoindolin-2-ylidene)acetonitrile **3b**

The product (222 mg, 0.7 mmol, 70% yield) was obtained as a dark-red powder (mp 300–301 °C) from 161 mg (1 mmol) of 5-methylisatin **1b** and 174 mg (1 mmol) of 2-(benzo[*d*]thiazol-2-yl)acetonitrile **2a**. **IR (KBr):** 3433, 2204, 1718, 1596.^**1**^**H NMR (400 MHz, DMSO-*****d***_**6**_**)** δ 2.33 (s, 3H), 7.40 (d, *J* = 8.2 Hz, 1H), 7.47–7.53 (m, 2H), 7.54 (s, 1H), 7.65 (ddd, *J* = 8.2, 7.6, 1.1 Hz, 1H), 8.18 (d, *J* = 7.8 Hz, 1H), 8.20 (d, *J* = 7.7 Hz, 1H), 11.48 (s, 1H). ^**13**^**C{**^**1**^**H} NMR (101 MHz, DMSO-*****d***_**6**_**)** δ 20.6 (CH_3_), 79.7 (C), 114.3 (CH), 116.9 (C), 120.0 (C), 122.9 (CH), 123.1 (CH), 125.6 (CH), 126.3 (CH), 127.6 (CH), 133.1 (C), 134.3(C), 138.8 (CH), 143.9 (C), 150.0 (C), 154.0 (C), 163.5 (C), 185.1 (C). **HRMS** (ESI) calcd for C_18_H_12_N_3_OS (M + H^+^) 318.0701, found 318.0704. CCDC deposition number: 2176039

#### 4.2.5 (*Z*)-2-(Benzo[*d*]thiazol-2-yl)-2-(5-methoxy-3-oxoindolin-2-ylidene)acetonitrile **3c**

The product (290 mg, 0.87 mmol, 87% yield) was obtained as a dark-violet powder (mp 297–298 °C) from 177 mg (1 mmol) of isatin **1c** and 174 mg (1 mmol) of 2-(benzo[*d*]thiazol-2-yl)acetonitrile **2a**. **IR (KBr):** 3428, 2201, 1702, 1591, 1213. ^**1**^**H NMR (400 MHz, DMSO-*****d***_**6**_**)** δ 3.81 (s, 1H), 7.25 (d, *J* = 2.2 Hz, 1H), 7.28 (dd, *J* = 8.6, 2.5 Hz, 1H), 7.44 (d, *J* = 8.6 Hz, 1H), 7.52 (t, *J* = 7.5 Hz, 1H), 7.65 (t, *J* = 7.7 Hz, 1H), 8.20 (d, *J* = 8.0 Hz, 1H), 8.18 (d, *J* = 8.2 Hz, 1H), 11.42 (s, 1H). ^**13**^**C{**^**1**^**H} NMR (101 MHz, DMSO-*****d***_**6**_**)** δ 56.3 (CH_3_), 79.6 (C), 108.9 (CH), 115.5 (C), 117.0 (C), 120.4 (C), 122.9 (CH), 123.1 (CH), 125.2 (CH), 126.2 (CH), 127.6 (CH), 134.2 (CH), 144.1 (C), 146.2 (C), 154.1 (C), 156.2 (C), 163.5 (C), 185.1 (C). **HRMS** (ESI) calcd for C_18_H_12_N_3_O_2_S (M + H^+^) 334.0650, found 334.0647.

#### 4.2.6 (*Z*)-2-(Benzo[*d*]oxazol-2-yl)-2-(3-oxoindolin-2-ylidene)acetonitrile **4**

The product (221 mg, 0.77 mmol, 77% yield) was obtained as a dark-red powder (mp >300 °C) from 147 mg (1 mmol) of isatin **1a** and 158 mg (1 mmol) of 2-(benzo[*d*]oxazol-2-yl)acetonitrile **2b**. **IR (KBr):** 3435, 2214, 1719, 1596. ^**1**^**H NMR (400 MHz, DMSO-*****d***_**6**_**)** δ 7.16 (td, *J* = 7.4, 0.8 Hz, 1H, 5-H_Is_), 7.47–7.51 (m, 2H, 5,6-H_Bzo_), 7.52–7.55 (m, 1H, 7-H_Is_), 7.67 (td, *J* = 7.8, 1.3 Hz, 1H, 6-H_Is_), 7.72 (d, *J* = 7.5 Hz, 1H, 4-H_Is_), 7.83–7.90 (m, 2H, 4,7-H_Bzo_), δ 11.37 (s, 1H, NH). ^**13**^**C{**^**1**^**H} NMR (101 MHz, DMSO-*****d***_***6***_**)** δ 74.0 (C), 111.6 (CH), 114.5 (C), 114.6 (CH), 119.97 (C), 120.01 (CH), 123.8 (CH), 125.7 (CH), 126.0 (CH), 126.6 (CH), 138.3 (CH), 141.7 (C), 145.9 (C), 149.9 (C), 151.7 (C), 159.5 (C), 184.6 (C). **HRMS** (ESI) calcd for C_17_H_10_N_3_O_2_ (M + H^+^) 288.0773, found 288.0773. CCDC deposition number: 2176040.

### 4.3 p*K*_a_ Determination

The apparent ionization constant (p*K*_a_) values of the compounds **3b** and **4** were determined spectrophotometrically.^35^ Titration of 3.00 mL of 40 μmol·L^–1^ of each compound was performed in H_2_O/DMSO by adding small portions of 0.010–0.10 M HCl or NaOH solutions and recording the absorption spectrum after each addition. The solution volume has not changed by more than 10%.

The calculation of apparent ionization constants was carried out using the absorption data according to the Henderson–Hasselbalch equation
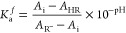
1where *A*_HR_ is the absorbance of the solution at the absorption maximum of the molecular form of the dye, *A*_R^–^_ is the absorbance of the solution at the absorption maximum of the ionic form of the dye, and *A*_i_ is the absorbance of the solution at the absorption maximum of the mixture of ionic and neutral forms, corresponding to a certain pH value, which was measured using a pH meter for each solution. The absorption maxima of the neutral and anionic forms of dyes are 530 and 680 nm (**3b**) and 515 and 645 nm (**4**), respectively.

### 4.4 Study of Complexation

The complexation study was realized as recommended in ref (36) using spectrophotometric titration according to the limited logarithm Bent and French method^30^ and the Molland method.^37^ For this purpose, two series of solutions were prepared. The first series of solutions contained equal amounts of Zn^2+^ (10 μmol·L^–1^ for **3b** and 5 μmol·L^–1^ for **4**), whereas the concentration of probes **3b** and **4** varied (10–90 μmol·L^–1^ and 0.5–81 μmol·L^–1^, respectively) upon the addition of the small portion of the corresponding probe. The second series of solutions contained equal amounts of probes **3b** (38 μmol·L^–1^) or **4** (9.9 μmol·L^–1^), whereas the concentration of Zn^2+^ varied (2–90 μmol·L^–1^ and 1–112 μmol·L^–1^, respectively). The absorption spectra of these solutions were recorded, and saturation curves Δ*A* as a function of *C*, μmol·L^–1^, were obtained, where Δ*A* = *A*_R_ – *A*_ZnR_ at a certain wavelength.

The molar composition of complexes (Zn*_m_*R*_n_*) was established using the formula *n*/*m* = (Δ*A*_max_^Zn^ × *C*_R_)/(Δ*A*_max_^R^ × *C*_Zn_), where *m* and *n* are the number of Zn^2+^ and the ligand attached to a metal ion consequently, respectively; Δ*A*_max_^Zn^ and Δ*A*_max_^R^ are absorption, corresponding to the saturation area; and *C*_R_ and *C*_Zn_ are the initial concentrations of the ligand and Zn^2+^ in solutions of each series, respectively.

The stability constants of the complexes were calculated using eq 2
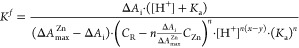
2where Δ*A*_i_ is the value of absorbance in the initial linear section of the saturation curves; *A*_max_^Zn^ is the value of absorbance in the saturation region; *C*_R_ is the concentration of the probe, which corresponds to Δ*A*_i_; *C*_Zn_ is the concentration of Zn^2+^; *n* is the number of the coordinated ligands; *x* is the number of protons capable of dissociation of dyes **3b** or **4** (*x* = 1); *y* is the number of protons detached from the dye (*y* = 1); and *K*_a_ is the ionization constant of dyes **3b** or **4**.

## Supporting Information Available

NMR spectra for all the ligands, photostability test data, absorption property data, deprotonation and complexation study data, and description of Zn^2+^ detection in the model solution of serum (PDF)

## Dedication

Dedicated to Prof. Olga A. Zaporozhets in memoriam.
