# Supplementary material for: 2-Azahetaryl-2-(oxoindolin-2-ylidene)acetonitriles as Colorimetric Probes for Zn: Synthesis and Optical Properties
Source: ACS Omega. 2022 Nov 16;7(47):42819–27. doi: 10.1021/acsomega.2c04747 (PMC9713784; doi:10.1021/acsomega.2c04747)
Supplement: Supplementary file 1 — ao2c04747_si_001.pdf [file ao2c04747_si_001.pdf]

Supporting Information

2-Azahetaryl-2-(oxoindolin-2-ylidenes)acetonitriles as colorimetric probes for Zn: synthesis and optical properties

Dedicated to Prof. Olga A. Zaporozhets in memoriam.

Vladyslav V. Shcherban[a*], Olena O. Kuleshova [a,b], Tetiana Ye. Keda [a*], Olga V. Khilya [a], Emmanuel Gras [b, 1], Yulian M. Volovenko [a]

[a] Faculty of Chemistry, Taras Shevchenko National University of Kyiv, Lva Tolstoho Street 12, Kyiv 01033, Ukraine

[b] Laboratoire de Chimie de Coordination, Centre National de la Recherche Scientifique, UPR8241, Université Fédérale Toulouse Midi-Pyrénées, 205, Route de Narbonne, Toulouse F-31077, France

Corresponding authors

Vladyslav V. Shcherban: schcherban@gmail.com

Tetiana Ye. Keda: tetianakeda@knu.ua

[1] LHFA CNRS - UMR 5069, Université Paul Sabatier, 118, route de Narbonne, 31062 Toulouse Cedex 9

Table of Contents

1. ^{1}H, ^{13}C Data 3

2. Photostability test 16

3. Absorption properties of probe 3a-c, 4 and 3b-Zn^{2+}, 4-Zn^{2+} in H_2O/DMSO solutions. 16

4. Deprotonation studies 20

5. Complexation studies 22

6. Selectivity studies 23

7. Detecting of Zn^{2+} in serum model solution 24

1. **[1]H, [13]C Data**

Figure S1. ^{1}H-NMR spectra of the compound (*Z*)-2-(benzo[*d*]thiazol-2-yl)-2-(3-oxoindolin-2-ylidene)acetonitrile (**3a**), Solvent: DSMO-d_6

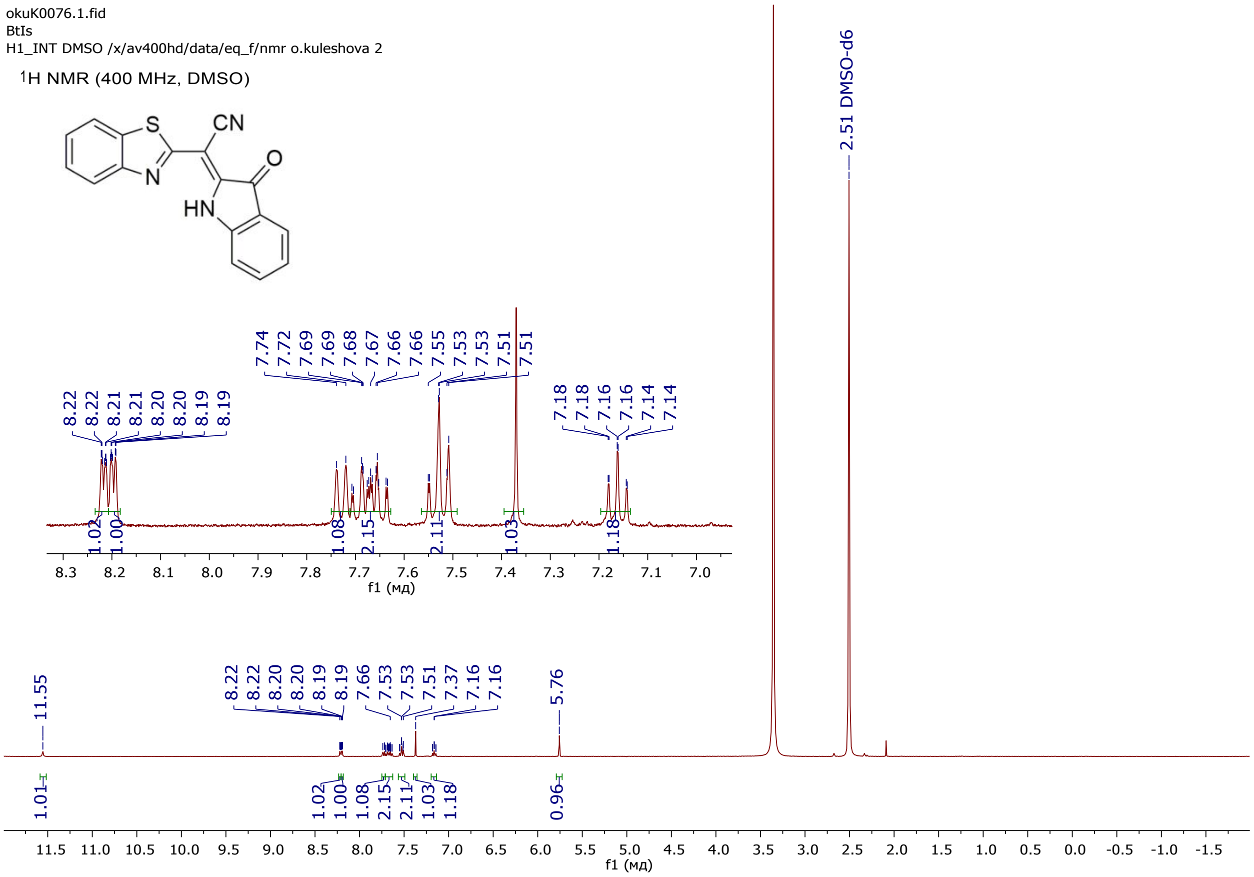

Figure S2. ^{13}C-NMR spectra of the compound (*Z*)-2-(benzo[*d*]thiazol-2-yl)-2-(3-oxoindolin-2-ylidene)acetonitrile (**3a**)

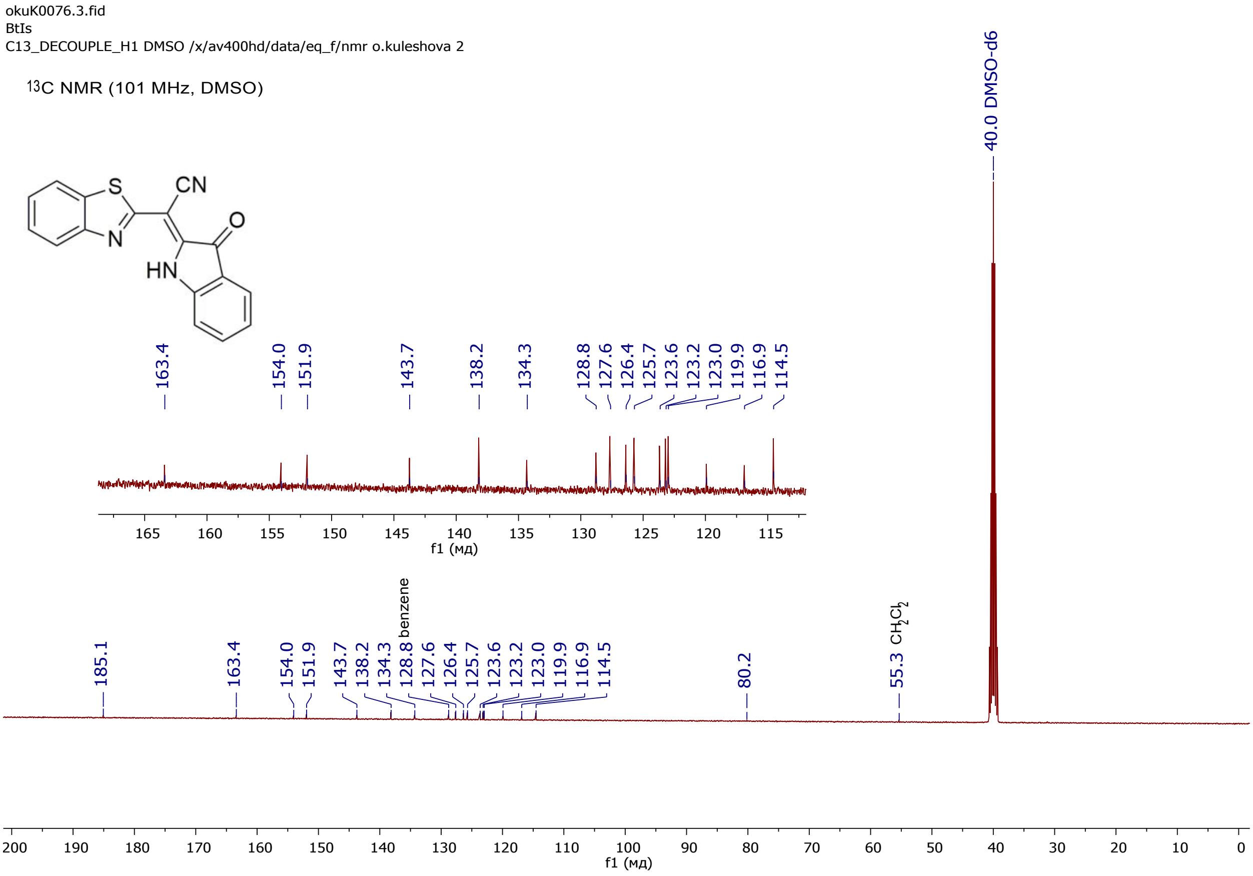

Figure S3. ^{1}H-NMR spectra of the compound (*Z*)-2-(benzo[*d*]thiazol-2-yl)-2-(5-methyl-3-oxoindolin-2-ylidene)acetonitrile (**3b**), Solvent: DMSO-d_6

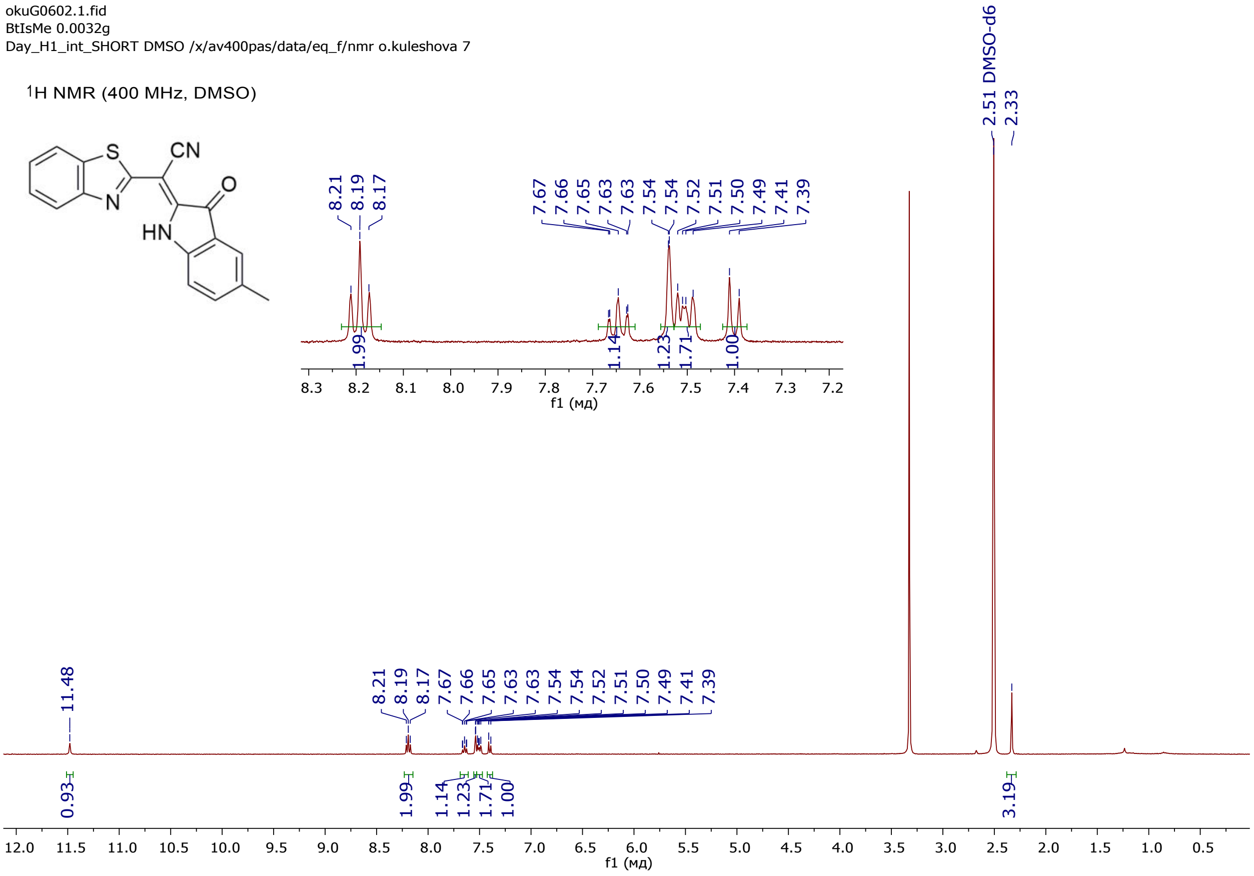

Figure S4. ^{13}C-NMR spectra of the compound (*Z*)-2-(benzo[*d*]thiazol-2-yl)-2-(5-methyl-3-oxoindolin-2-ylidene)acetonitrile (**3b**), Solvent: DMSO-d_6

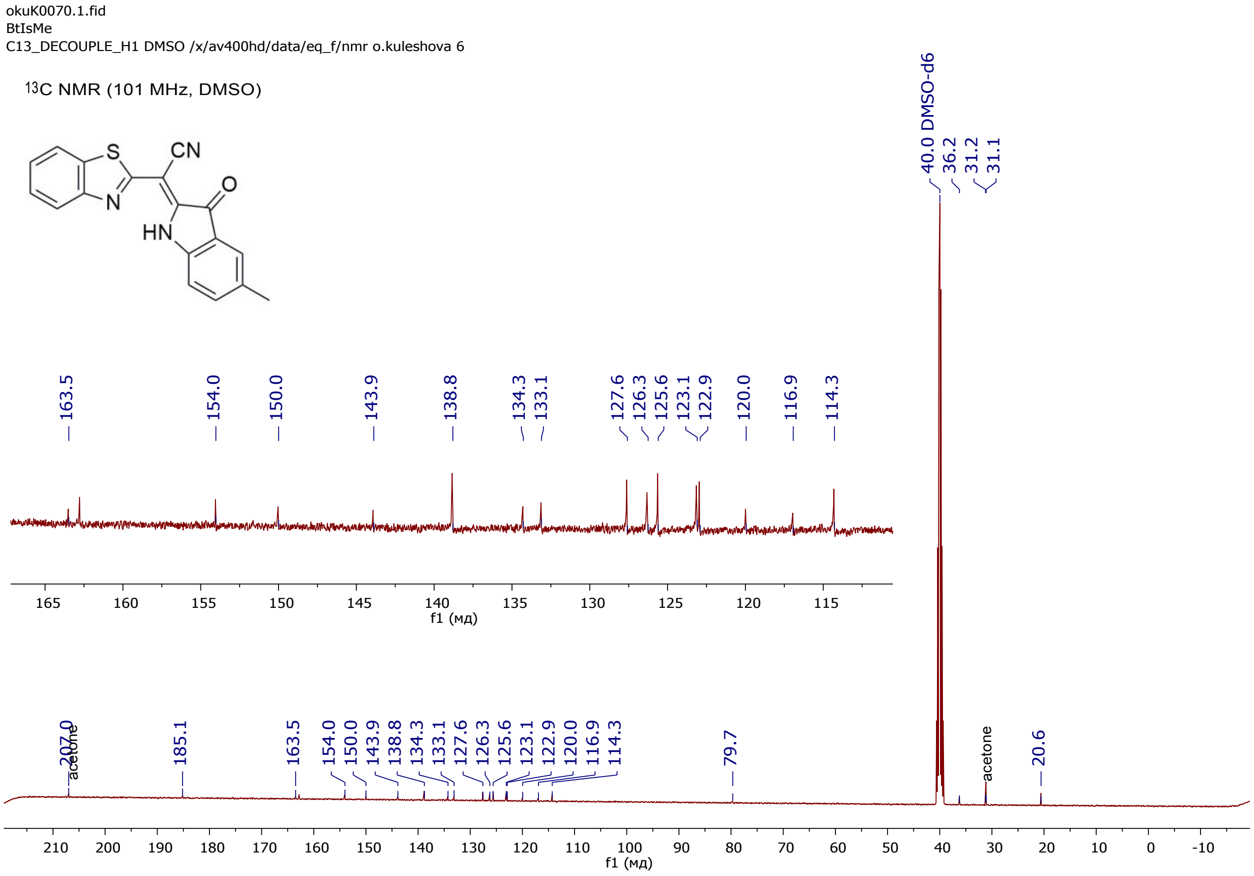

Figure S5. ^{1}H-NMR spectra of the compound (*Z*)-2-(benzo[*d*]thiazol-2-yl)-2-(5-methyl-3-oxoindolin-2-ylidene)acetonitrile (**3b**), Solvent CD_2Cl_2

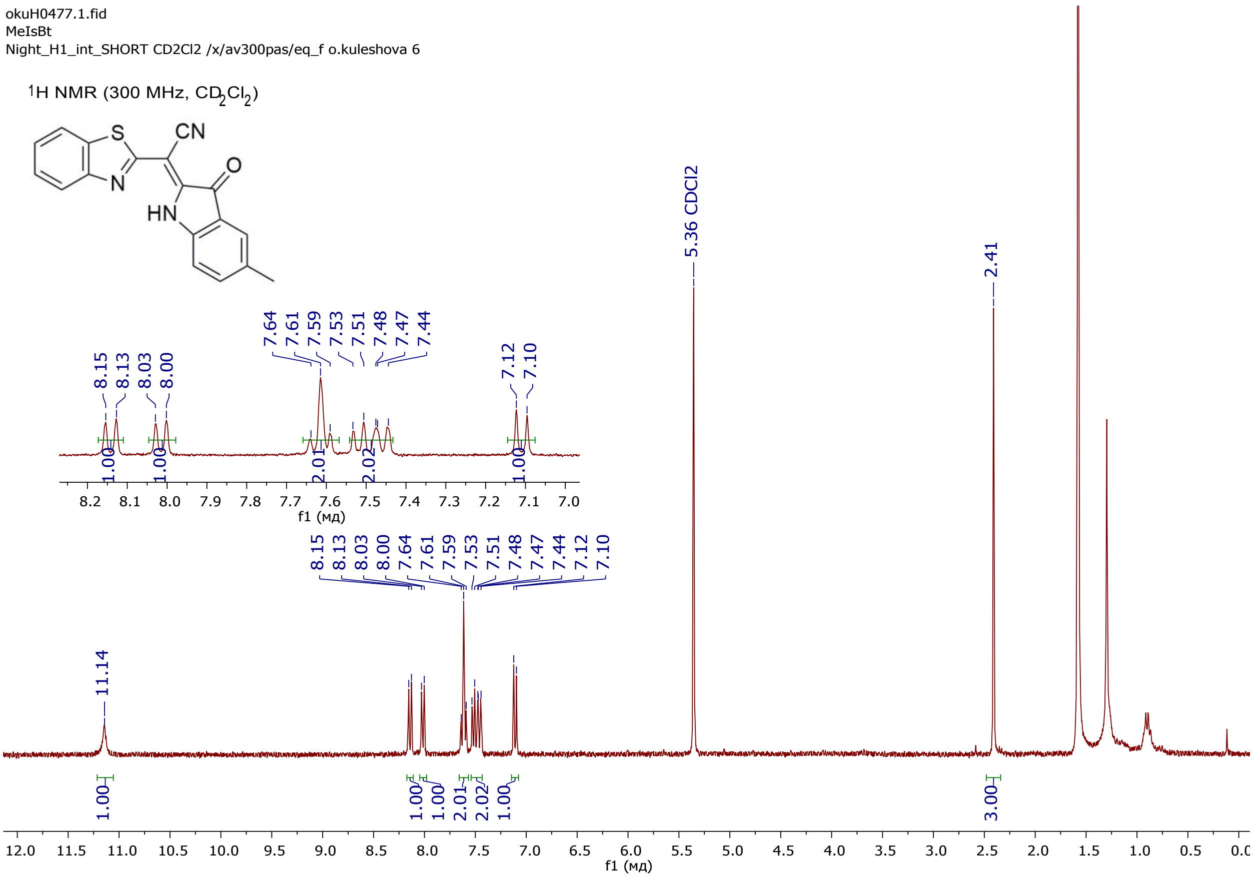

Figure S6. ^{1}H-NMR spectra of the compound (Z)-2-(benzo[d]thiazol-2-yl)-2-(5-methoxy-3-oxoindolin-2-ylidene)acetonitrile (**3c**), Solvent: DMSO-d_6

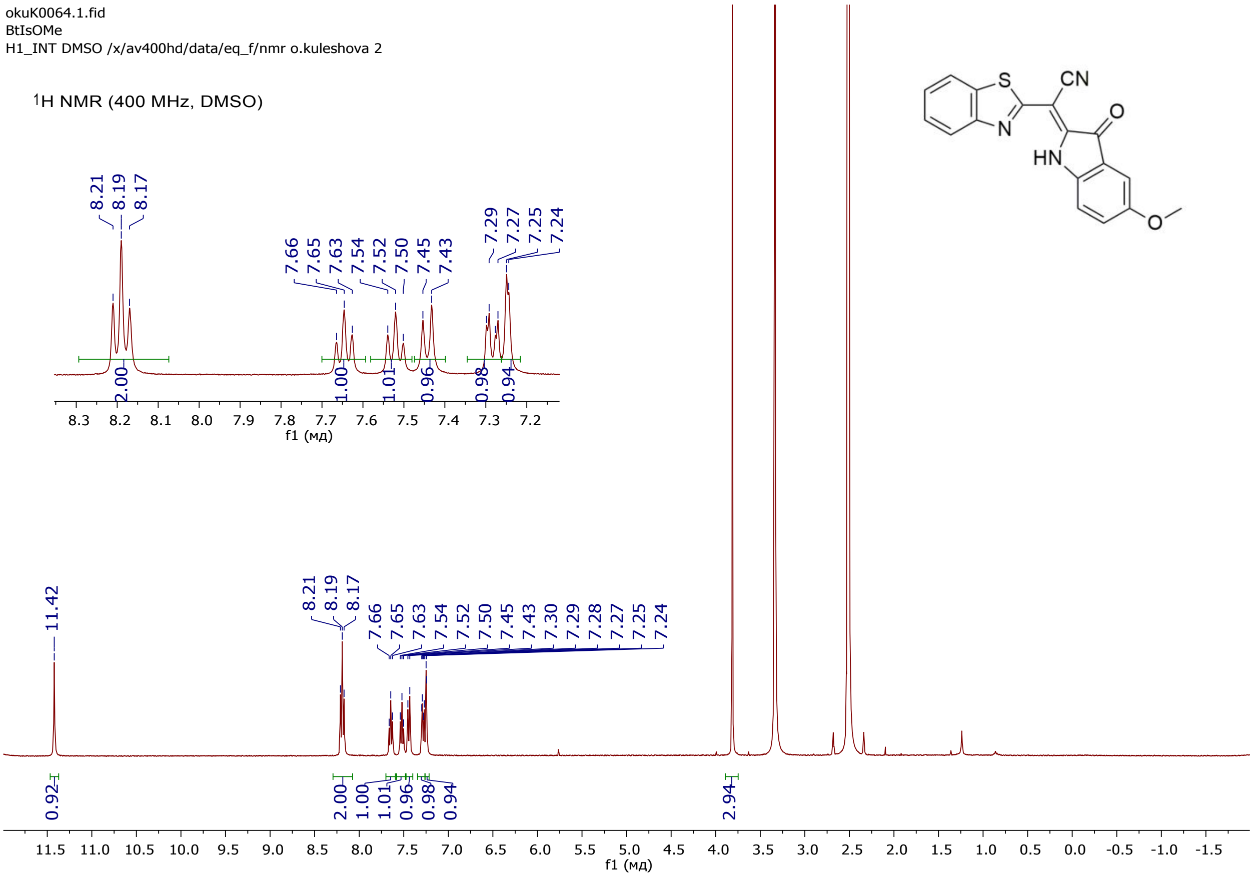

Figure S7. ^{13}C-NMR spectra of the compound (*Z*)-2-(benzo[*d*]thiazol-2-yl)-2-(5-methoxy-3-oxoindolin-2-ylidene)acetonitrile (**3c**), Solvent: DMSO-d_6

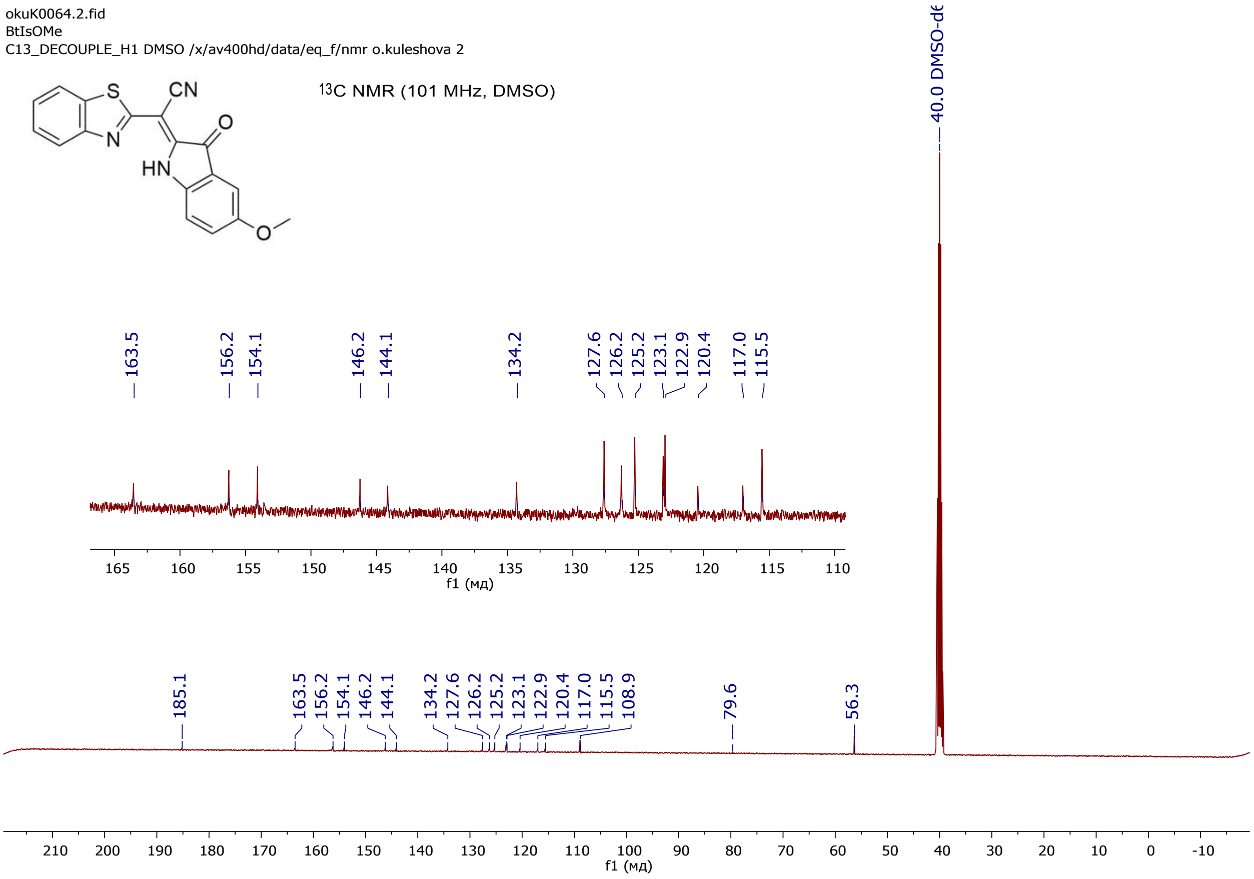

Figure S8. ^{1}H-NMR spectra of the compound (*Z*)-2-(benzo[*d*]oxazol-2-yl)-2-(3-oxoindolin-2-ylidene)acetonitrile (**4**), Solvent: DMSO-d_6

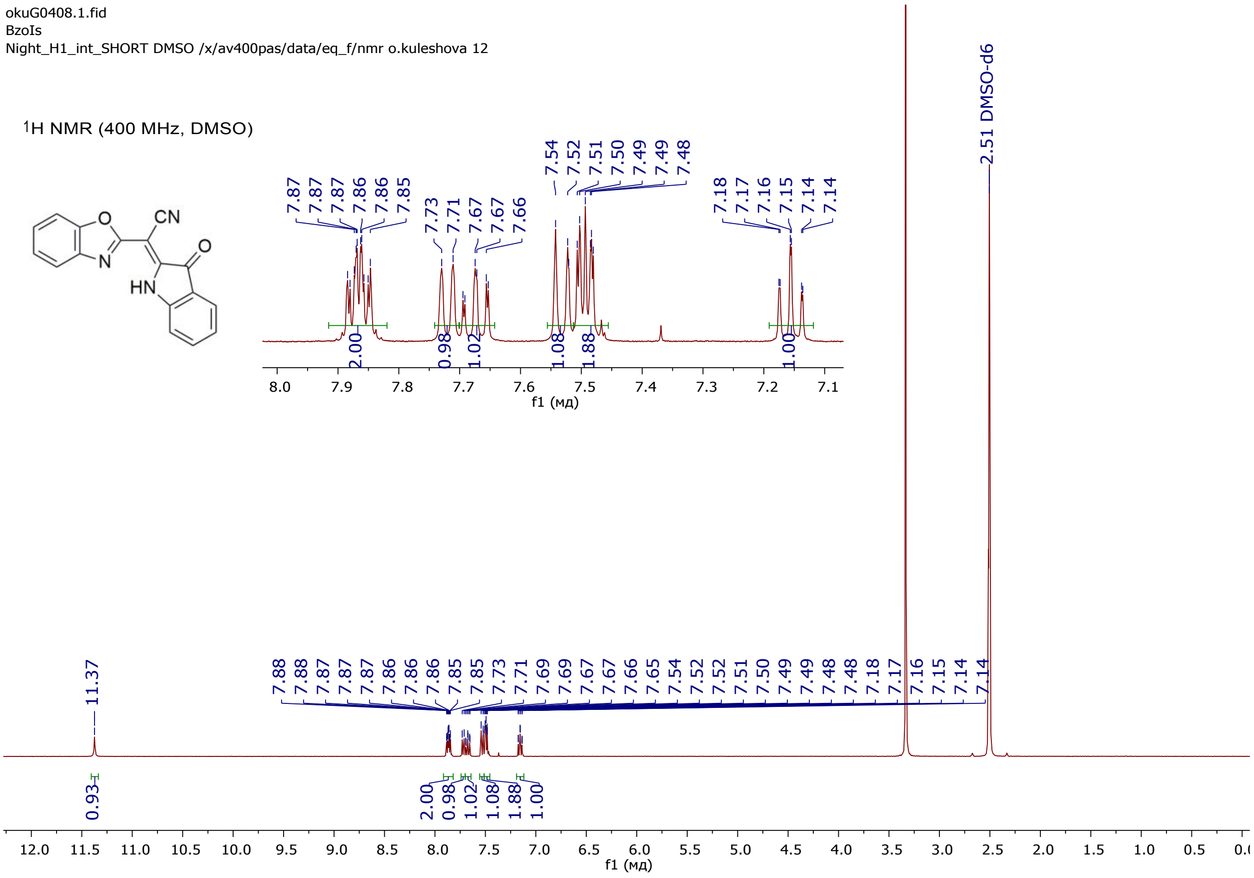

Figure S9. ^{13}C-NMR spectra of the compound (*Z*)-2-(benzo[*d*]oxazol-2-yl)-2-(3-oxoindolin-2-ylidene)acetonitrile (**4**), Solvent: DMSO-d_6

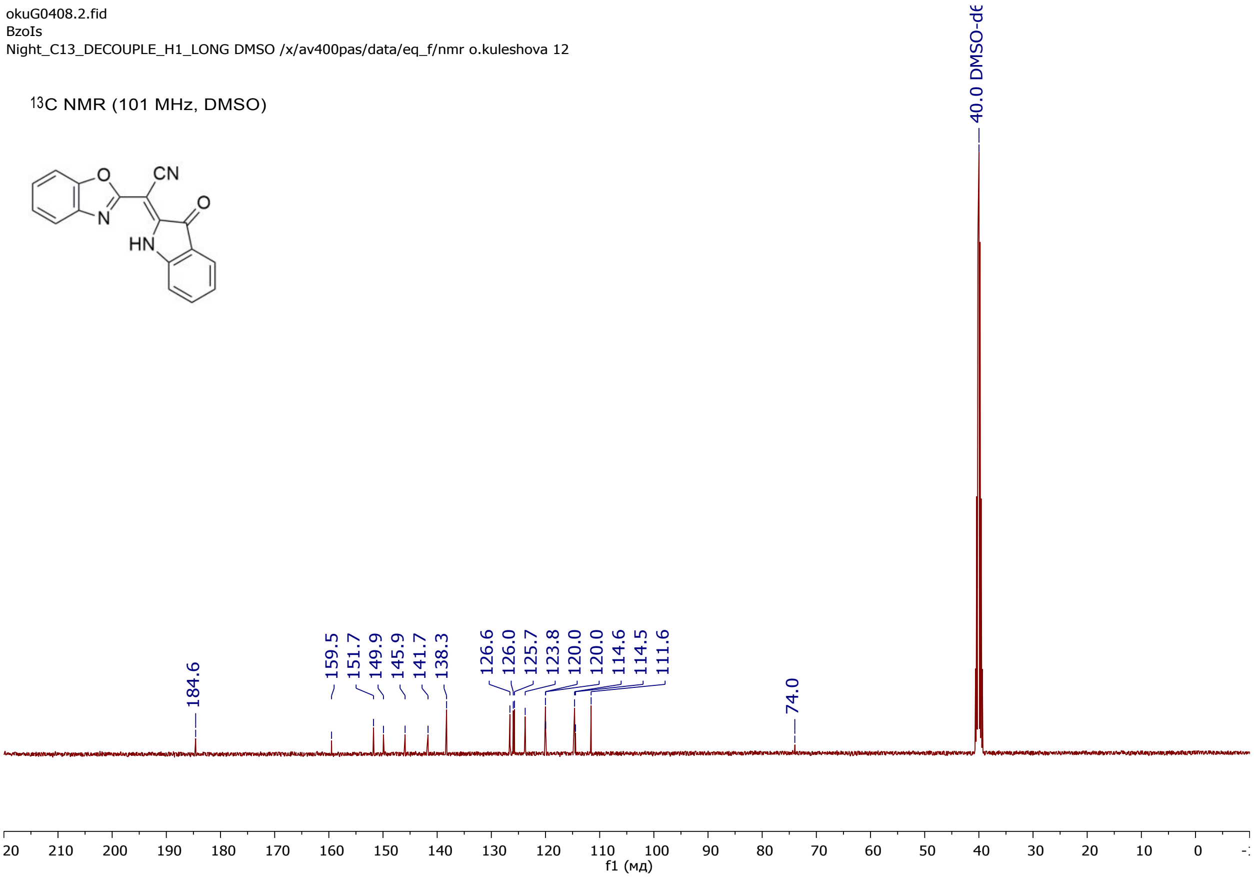

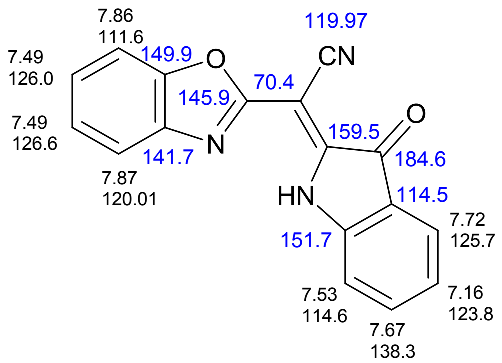

Figure S10. The NMR chemical shifts of **4** found by 2D experiments (COSY, HMQC, HMBC)

Table S1. The full list of heteronuclear correlations 1H–13C, founded for compound **4** in HMQC and HMBC spectra (Solvent: DMSO-d_6)

^1H	Position of cross peaks in ^{13}C dimension	
	HMQC	HMBC
7.16	123.8	114.6, 125.7, 138.2, 151.7, 184.6
7.49	126.0	120.01, 149.9
7.49	126.6	111.6, 141.7
7.53	114.6	123.8, 151.7
7.67	138.3	151.7, 125.7, 114.6
7.72	125.7	138.3, 151.7, 184.6
7.86	111.6	141.7, 126.6
7.87	120.01	149.9, 126.0
11.37	–	119.97, 145.9, 151.7, 184.6

Figure S11. COSY NMR spectrum of the compound **4,** Solvent: DMSO-d_6

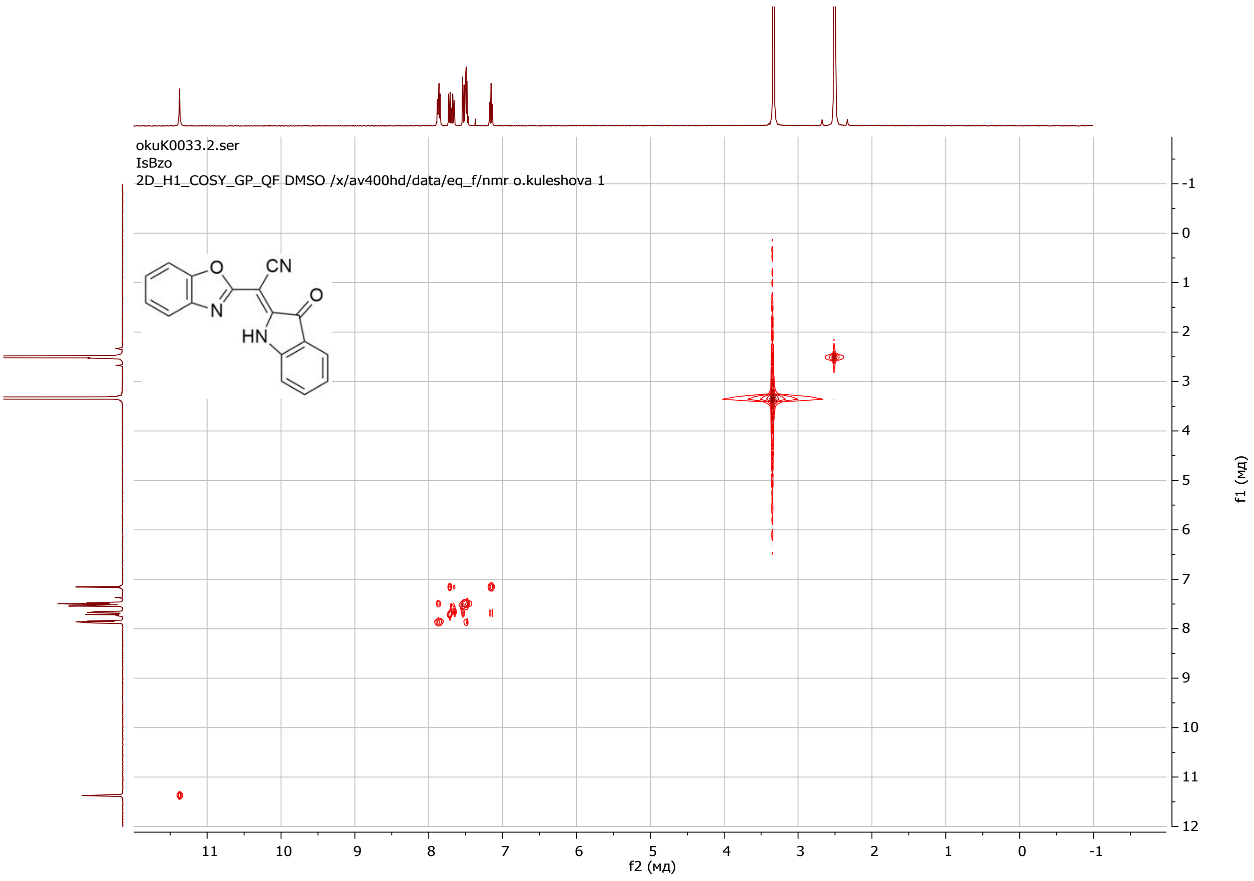

Figure S12. HMQC NMR spectrum of the compound **4**, Solvent: DMSO-d_6

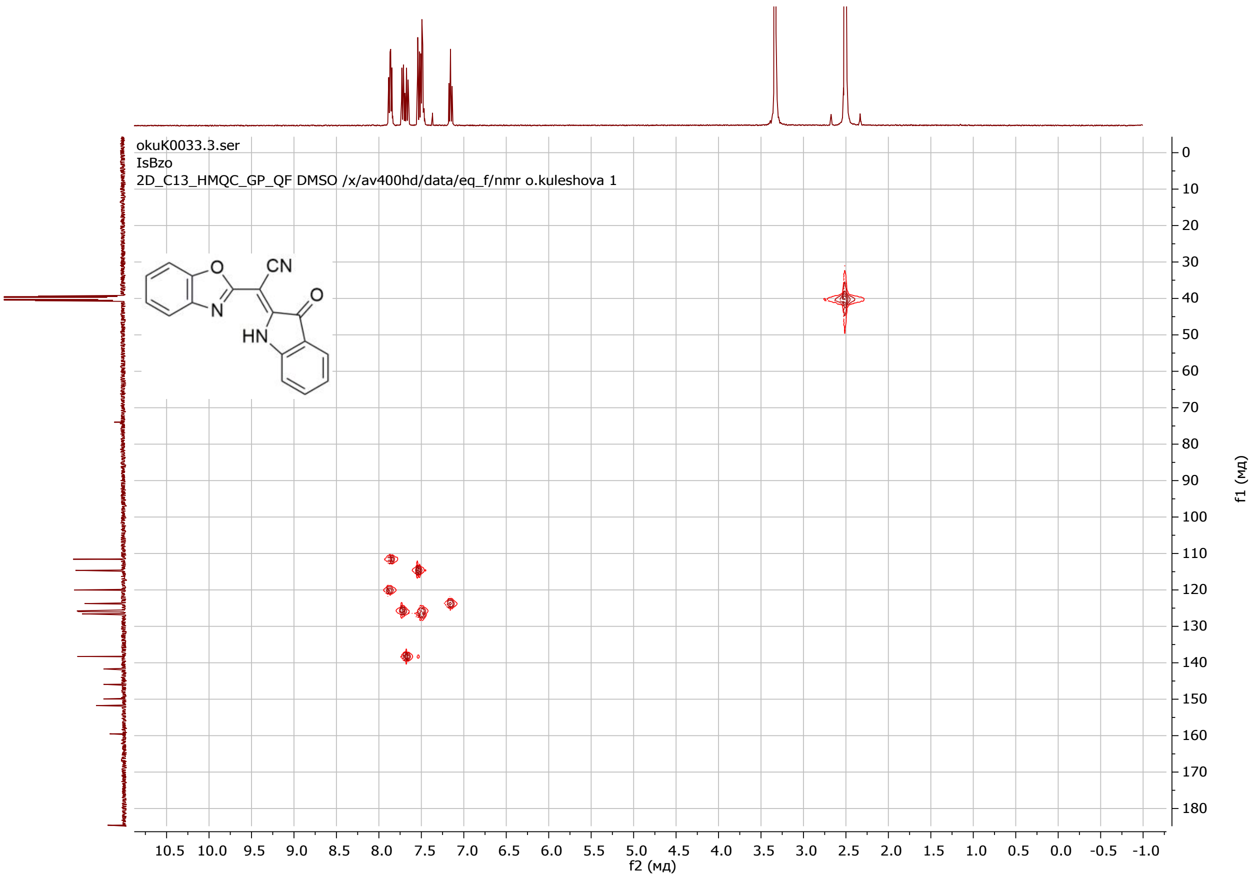

Figure S13. HMBC NMR spectrum of the compound **4,** Solvent: DMSO-d_6

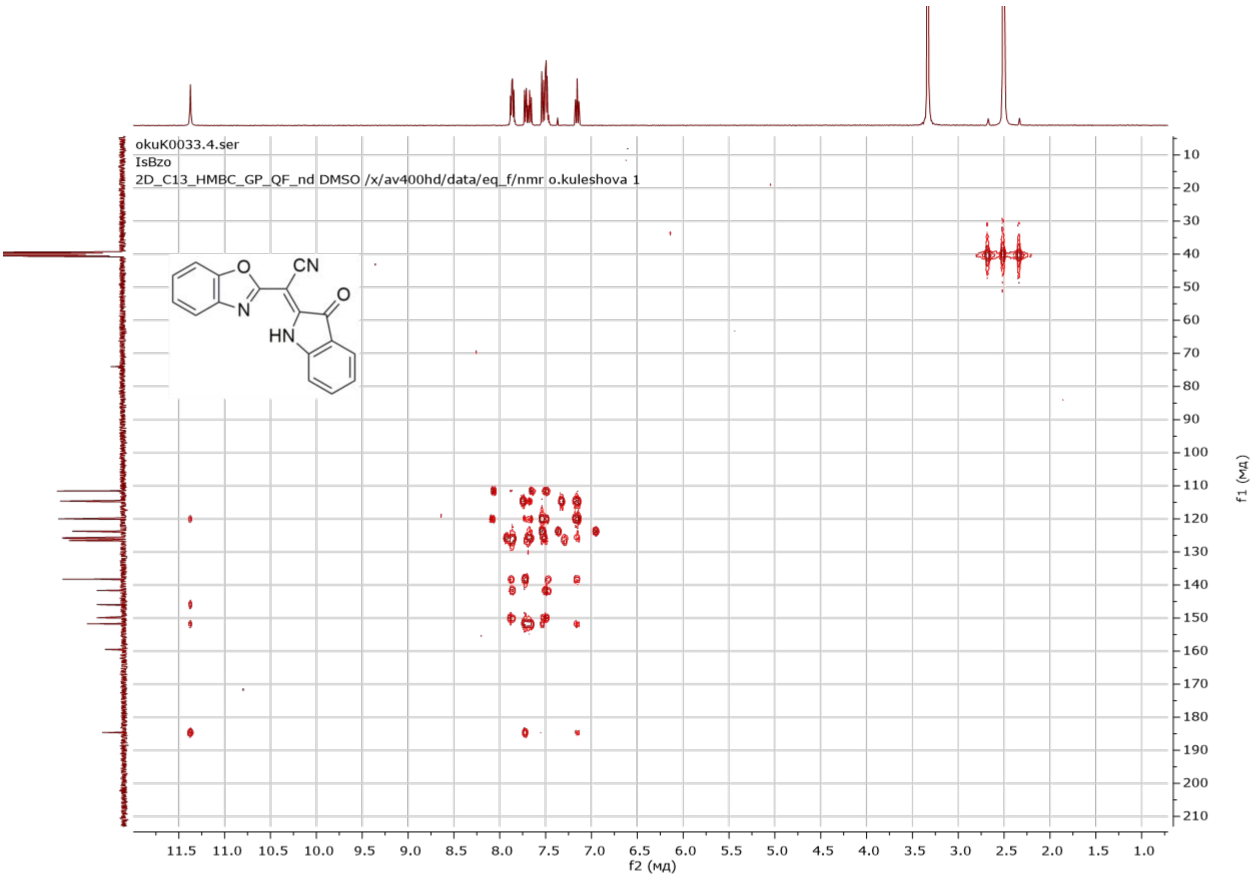

2. Photostability test

The photostability test was performed for the studied dyes in DMSO as a control for the absorbance of a diluted solution of the appropriate concentration. Dye **3b** was used as an example.

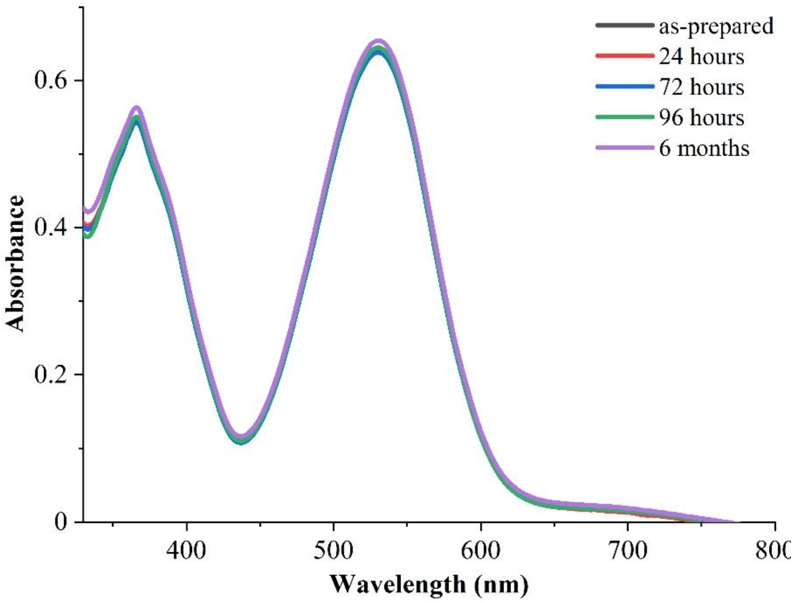

Figure S14. Absorption spectra of dye **3b** in DMSO as-prepared and after 24, 72, 96 h and 6 months of storage. C_{3b}= 50 μmol·L^{-1}

3. Absorption properties of probe 3a-c, 4 and 3b-Zn^{2+}, 4-Zn^{2+} in H_2O/DMSO solutions.

The molar absorption coefficient (ε, mol^{-1}Lcm^{-1}) was calculated according the Beer-Lambert law using value of the absorbance of solution at the appropriate wavelength of UV-Vis spectra.

Table S2. Molar absorption coefficient of dyes **3a**, **3b** and **4** in DMSO and H_2O/DMSO

Dye	λ_{max}, nm	ε_{max} (DMSO), 10^4	ε_{max}, 10^4			$\Delta\varepsilon_{max}$,%		
			H_2O/DMSO, v/v					
			1/11	1/5	1/2	1/11	1/5	1/2
**3a**	520	1.48	1.59	1.59	**0.85**	7.4	7.4	**-42.6**
**3b**	530	1.38	1.46	1.43	1.44	5.8	3.6	4.3
**4**	515	1.66	1.59	1.59	1.53	-4.2	-4.2	-7.8

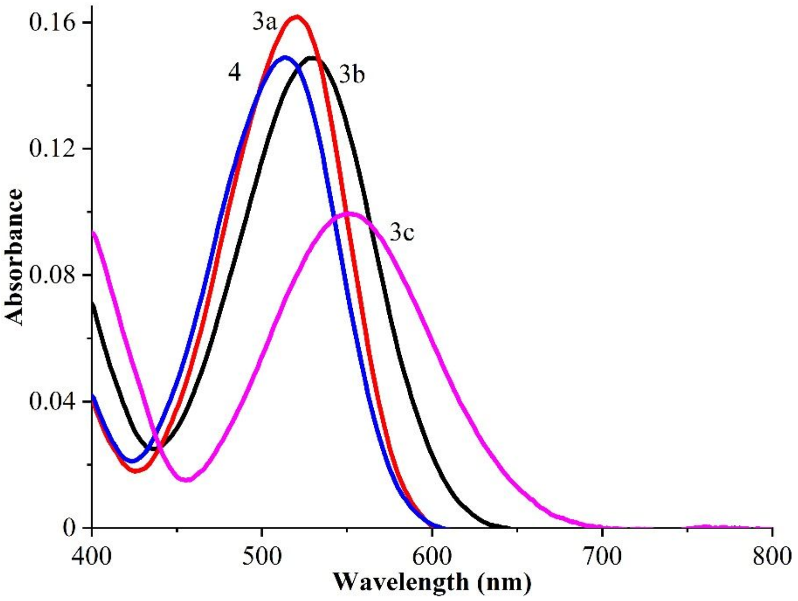

Figure S15. Absorption spectra of dye **3a-c** and **4** in H_2O/ DMSO (1/5, v/v). C = 10 μmol·L^{-1}

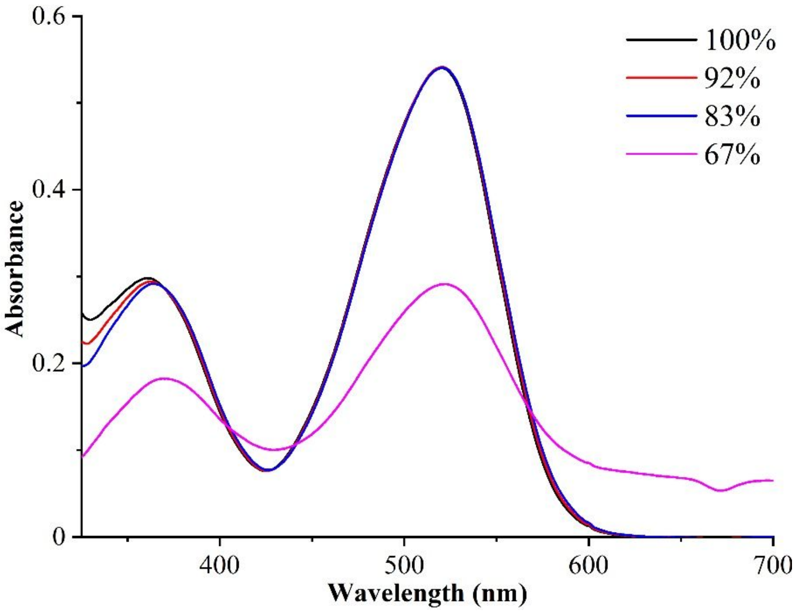

Figure S16. Absorption spectra of dye **3a** in DMSO and H_2O/ DMSO. % (v/v) DMSO is shown in the inset. C_{3a} = 34 μmol·L^{-1}

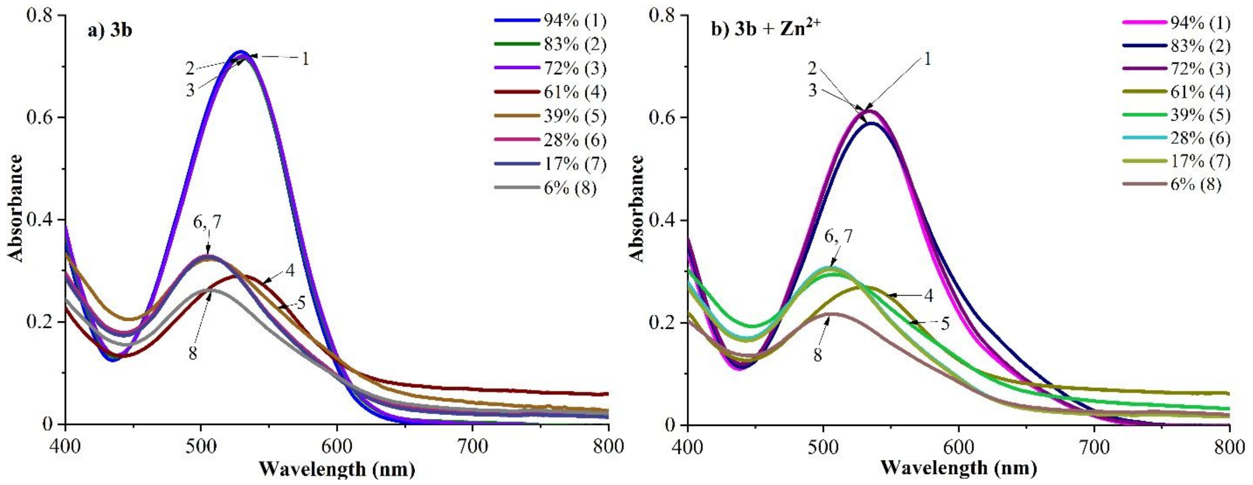

Figure S17. Absorption spectra of dye **3b** in Tris-buffer (pH 7.2) / DMSO without (a) and in the presence of **50** μmol·L^{-1} Zn^{2+} (b). % (v/v) DMSO is shown in the insets. $C_{\mathbf{3b}}$ = **50** μmol·L^{-1}.

Table S3. The influence of water on the absorbance of dye **3b** and **3b-Zn^{2+}** in solutions with different ratio H_2O (Tris-buffer, pH 7.2) / DMSO. $C_{\mathbf{3b}}$ = C(Zn^{2+}) = **50** μmol·L^{-1}. Sample volume – 3.00 ml. ΔA_{640}= A_{640}(3b) - A_{640}(3b+Zn^{2+})

№	V (DMSO), ml	V (H_2O), ml	% DMSO	A_{640}		ΔA_{640}
				3b	3b+Zn^{2+}	
1	2.83	0.17	94	0.0088	0.102	0.0932
2	**2.50**	**0.50**	**83**	**0.0154**	**0.1405**	**0.1251**
3	2.17	0.83	72	0.0159	0.1082	0.0923
4	1.84	1.16	61	0.081	0.0854	0.0044
5	1.17	1.83	39	0.0673	0.0727	0.0054
6	0.85	2.15	28	0.0389	0.0389	0
7	0.50	2.50	17	0.0349	0.0372	0.0023
8	0.18	2.82	6	0.0452	0.0408	-0.0044

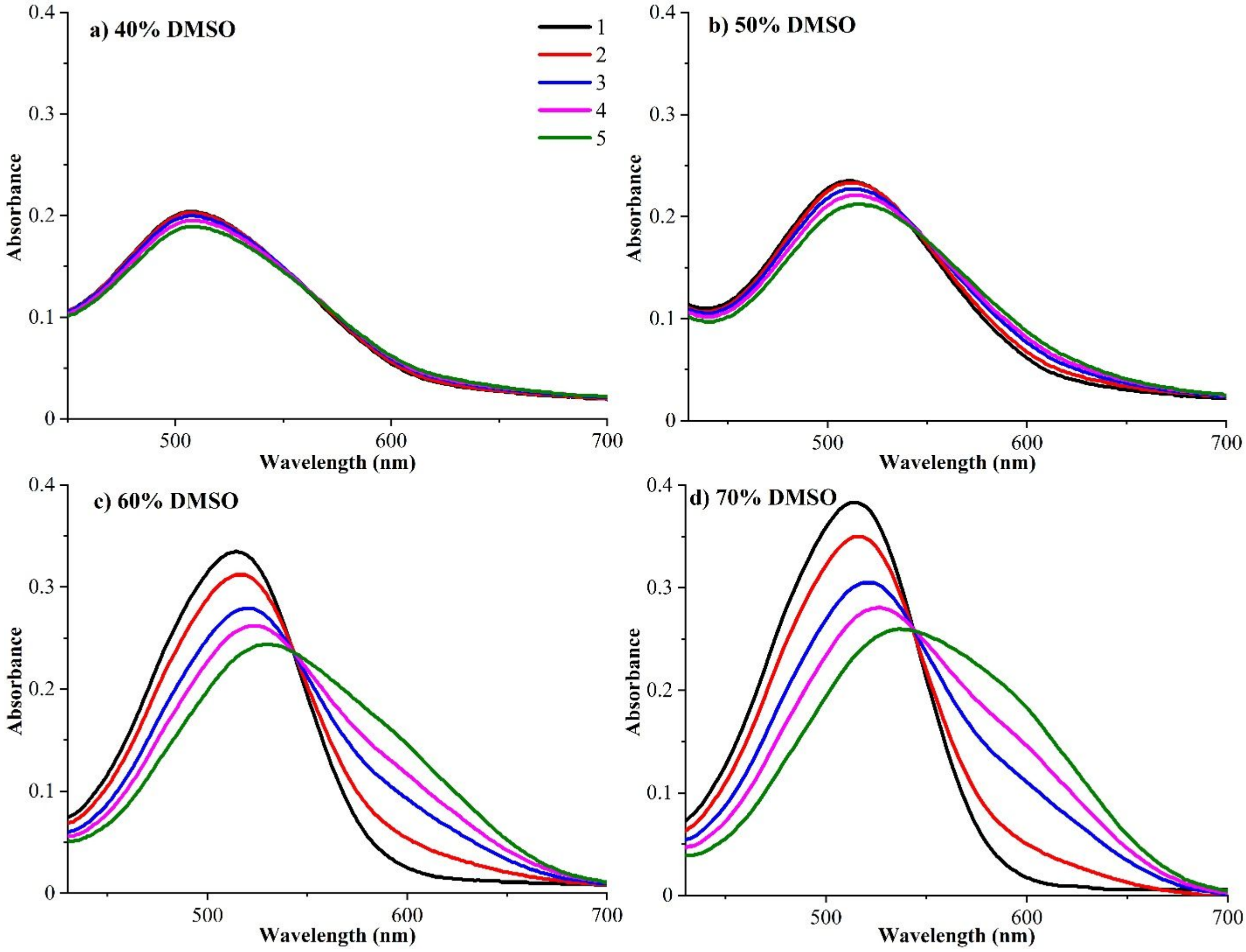

Figure S18. Absorption spectra of dye **4** without (1) and in the presence of different amount of Zn^{2+} (2-5), C_4 = 25.0 µmol·L^{-1}, Tris-buffer (pH 7.2)/DMSO with different %, v/v of DMSO. C(Zn^{2+}), µmol·L^{-1} : 0 (1), 5.0 (2), 15.0 (3), 24.0 (4), 43.0 (5).

4. Deprotonation studies

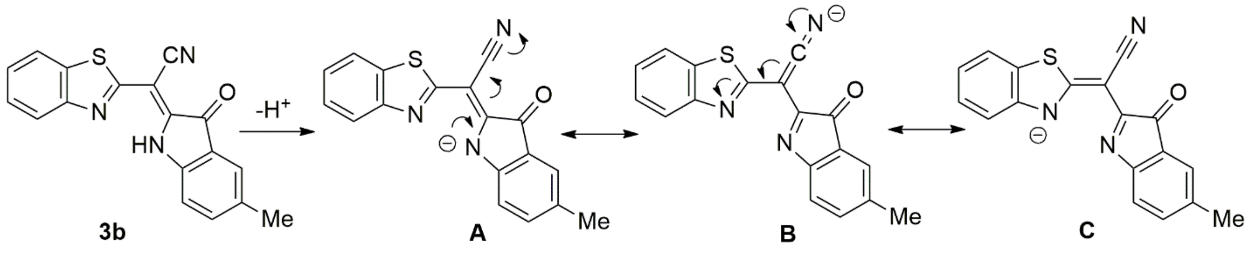

Scheme S1. Possible way of negative charge delocalization upon deprotonation of **3b**

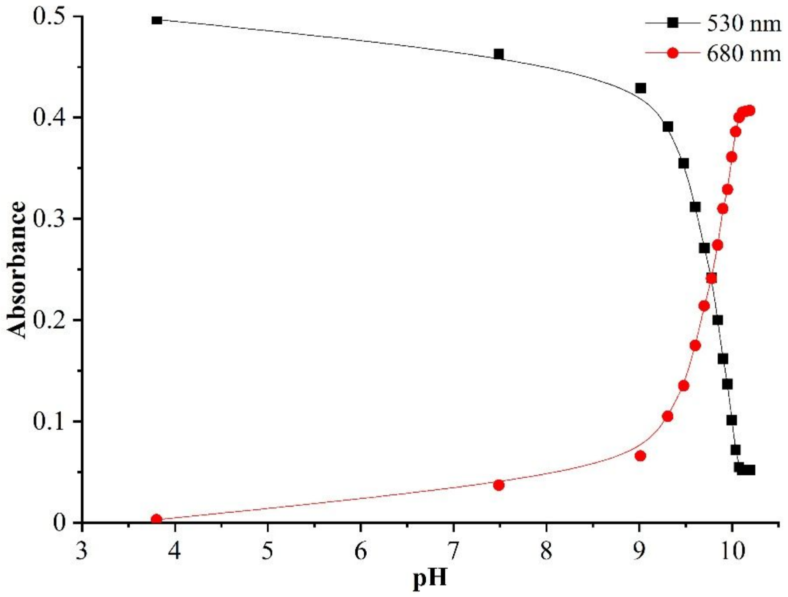

Figure S19. Spectrophotometric titration plots at the absorption maxima of dye **3b** in H_2O/DMSO (1/5, v/v). pH corresponds to the calculated concentration of H^+ in the aqueous part of the sample (0.5 ml). Black line corresponds to the absorption of neutral form; red line – absorption of anionic form of the dye.

Table S4. Calculation data of the ionization constant of dye **3b** in H_2O/DMSO solutions.

№	pH	λ_1 = 530 nm		λ_2 = 680 nm	
		A	pKa	A	pKa
1	10.15	0.463	-	0.037	-
2	12.70	0.429	13.44	0.066	13.43
3	13.19	0.391	13.69	0.105	13.66
4	1348	0.355	13.81	0.135	13.79
5	13.69	0.312	13.83	0.175	13.81
6	13.85	0.271	13.83	0.214	13.81
7	13.98	0.242	13.85	0.241	13.82
8	14.09	0.200	13.79	0.274	13.78
9	14.18	0.162	13.70	0.310	13.68
10	14.27	0.137	13.64	0.329	13.64
11	14.34	0.101	13.44	0.361	13.44
12	14.41	0.072	-	0.386	-
13	14.48	0.055	-	0.400	-

Table S5. Calculation data of the ionization constant of dye **4** in H_2O/DMSO solutions.

№	pH	λ_1 = 515 nm		λ_2 = 645 nm	
		A	pKa	A	pKa
1	13.43	0.573	-	0.003	-
2	13.76	0.565	-	0.003	-
3	14.17	0.561	15.69	0.014	15.77
4	14.30	0.547	15.53	0.015	15.86
5	14.52	0.512	15.37	0.044	15.48
6	14.61	0.477	15.23	0.069	15.33
7	14.69	0.428	15.08	0.113	15.12
8	14.76	0.335	14.81	0.188	14.84
9	14.82	0.245	14.56	0.264	14.57
10	14.88	0.153	-	0.345	-
11	14.93	0.107	-	0.378	-

5. Complexation studies

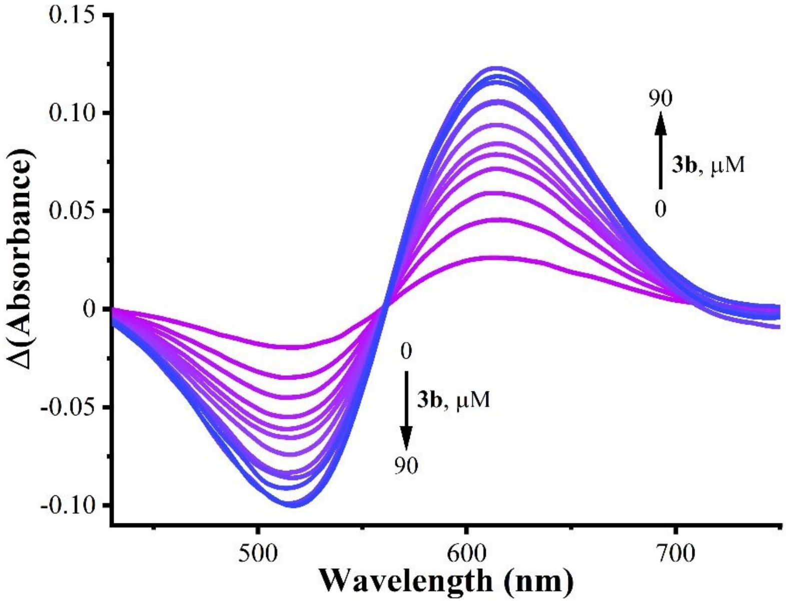

Figure S20. Absorption spectra of different concentrations probe **3b** solutions in the presence of Zn^{2+}, $C(Zn^{2+})$ = 10.0 μmol·L^{-1}, Tris-buffer (pH 7.2)/DMSO (1/5, v/v)

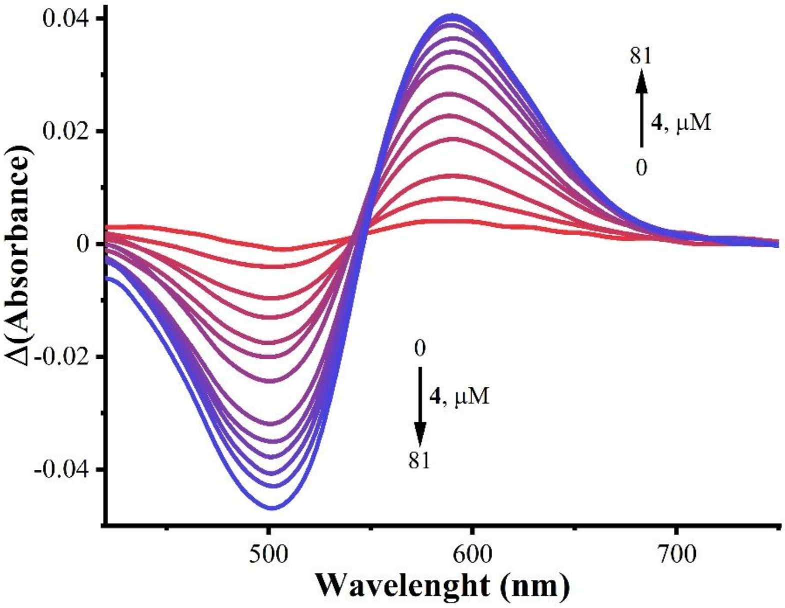

Figure S21. Absorption spectra of different concentrations probe **4** solutions in the presence of Zn^{2+}, $C(Zn^{2+})$ = 4.8 μmol·L^{-1}, Tris-buffer (pH 7.2)/DMSO (1/3, v/v).

Table S6. Calculation data of the affinity constant of the Zn^{2+} complex with probe **3b** by the Bent-French method

№	C_{3b}, μmol·L^{-1}	ΔA_{600}	lgK^f
1	2.04	0.010	10.26
2	2.63	0.013	10.28
3	3.13	0.015	10.25
4	3.78	0.018	10.26
5	4.61	0.022	10.28
6	5.12	0.025	10.31
7	5.88	0.027	10.28
8	6.36	0.029	10.28

Table S7. Calculation data of the affinity constant of the Zn^{2+} complex with probe **4** by the Bent-French method

№	C_4, μmol·L^{-1}	ΔA_{590}	lgK^f
1	1.1	0.009	12.77
2	1.4	0.010	12.69
3	1.7	0.011	12.55
4	2.0	0.012	12.49
5	2.5	0.013	12.41
6	3.0	0.014	12.32
7	3.4	0.015	12.28
8	4.0	0.016	12.22

6. Selectivity studies

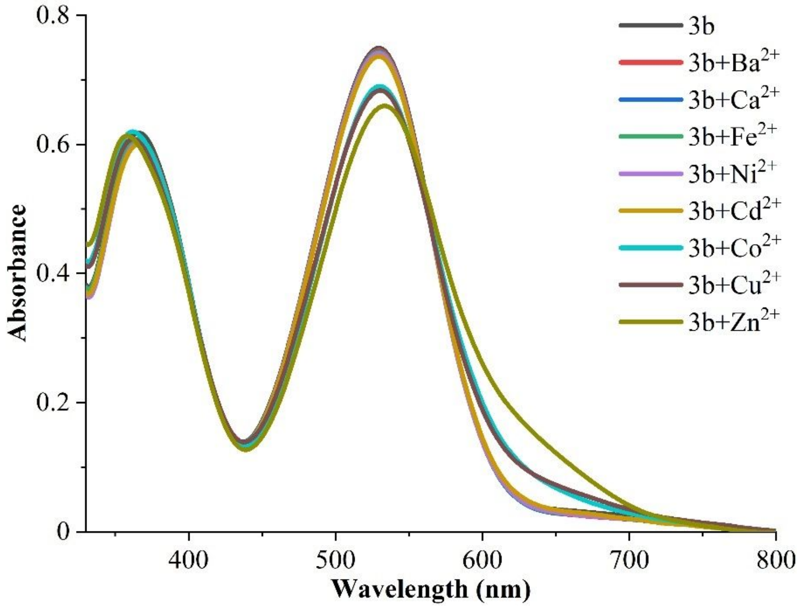

Figure S22. Absorption spectra of probe **3b** without and in the presence of different ions after 60 min prepared of solution. Tris-buffer (pH 7.2)/DMSO (1/5, v/v);
C_{3b} = 50.0 μmol·L^{-1}; Concentration, μmol·L^{-1}: 100 (Ba^{2+}, Ca^{2+}); 10 (Fe^{2+}, Ni^{2+}, Cd^{2+}, Co^{2+}, Cu^{2+}, Zn^{2+}).

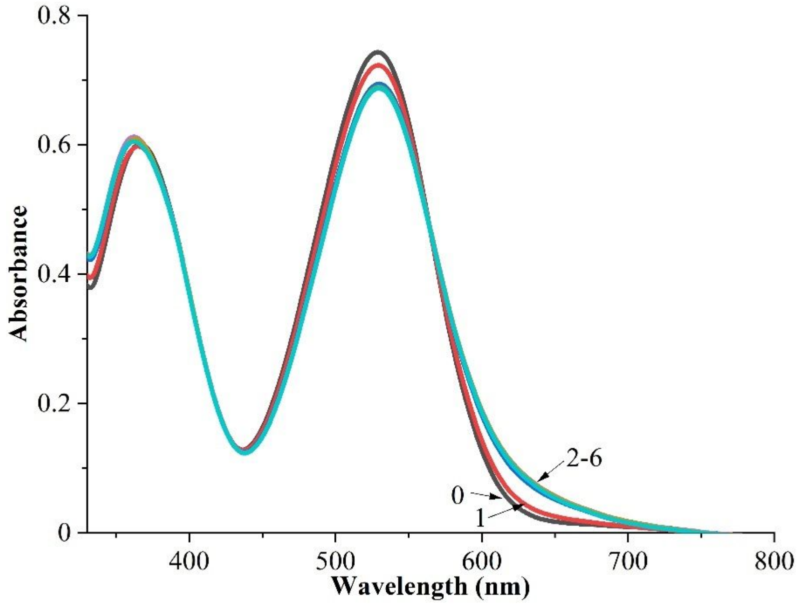

Figure S23. Absorption spectra of probe **3b** without (0) and in the presence of 10 μmol·L^{-1} Co^{2+} after 15 min (1), 30-60 min (2-6) of prepared. C_{3b} = 50.0 μmol.L^{-1}, Tris-buffer (pH 7.2)/DMSO (1/5, v/v).

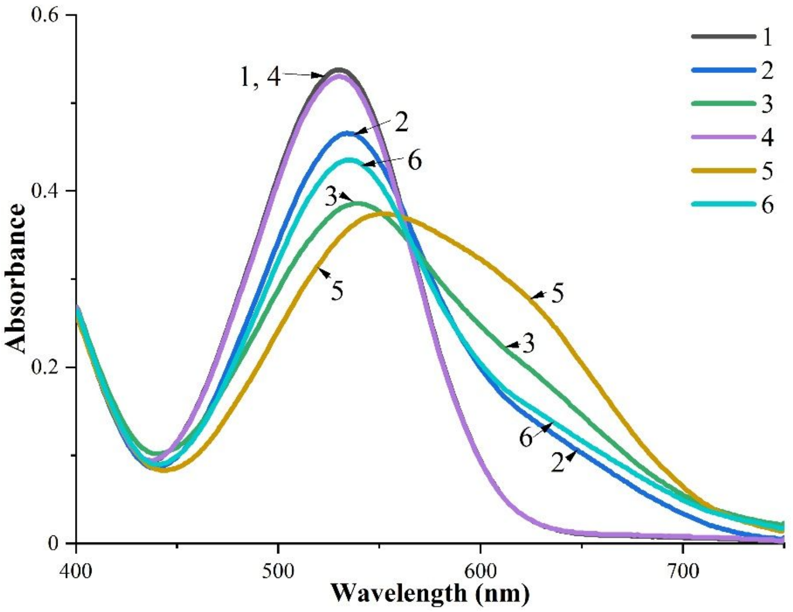

Figure S24. Absorption spectra of probe **3b** (1); **3b** with Zn^{2+} in the presence of $Na_2S_2O_3$ (2); **3b** with Cu^{2+} without (3) and in the presence of $Na_2S_2O_3$ (4); **3b** with Zn^{2+} and Cu^{2+} without (5) and in the presence of $Na_2S_2O_3$ (6). Tris-buffer (pH 7.2)/DMSO (1/5, v/v); C_{3b} = 40.0 μmol·L^{-1}; Concentration of Zn^{2+} – 9.0 μmol·L^{-1}; Cu^{2+} – 20.0 μmol·L^{-1}; $Na_2S_2O_3$ – 1.0 mmol·L^{-1}.

The thiosulfate ion is a well-known masking agent for Cu^{2+} due to the formation of complex compounds, particularly in the determination of Zn^{2+} with EDTA in the presence of Cu^{2+}.[1,2] Figure S24 shows the successful masking of 20.0 μmol·l^{-1} Cu^{2+} by 1.0 mmol·l^{-1} $Na_2S_2O_3$ under optimal conditions for the complexation of probe **3b** with Zn^{2+}.

The initial 50-fold excess of thiosulfate ions allows us to predict the formation of $Cu(S_2O_3)_n^{-(2n-1)}$ anionic complexes with n=1-3 in the solution.[3-5]

7. Detecting of Zn^{2+} in serum model solution

The model solutions of 2 times diluted pre-treated blood serum with 5.0 and 10.0 μmol·L^{-1} Zn^{2+} were performed using Tris-buffer (pH=7.2) as a solvent. Concentration of components in each solution was as follows, mmol·L^{-1}: NaCl –120, KCl – 2.4, $CaCl_2$ – 1.5, $MgCl_2$ – 0.6, $CuSO_4$, $FeSO_4$ – 0.005, $Na_2S_2O_3$ - 1.0. Sample preparation: 2.5 ml of 40 μmol·L^{-1} of probe **3b** solution in DMSO was mixed with 0.5 ml of model solution. Spectrum of each sample in cuvette (1.00 cm) was recorded at 380-800 nm versus Tris-buffer (pH 7.2)/DMSO (1/5, v/v) as a blank solution. Figure S25 shows the absorption spectra. Concentration of Zn^{2+} in sample was calculated using equation: $\Delta A_{600}=(0.005\pm0.002)+(0.0111\pm0.0002)\cdot C_{Zn}$, μmol·L^{-1}, where $\Delta A_{600} = A^i_{600} - A_{600}$ **(3b).** The data obtained are listed in Table S8.

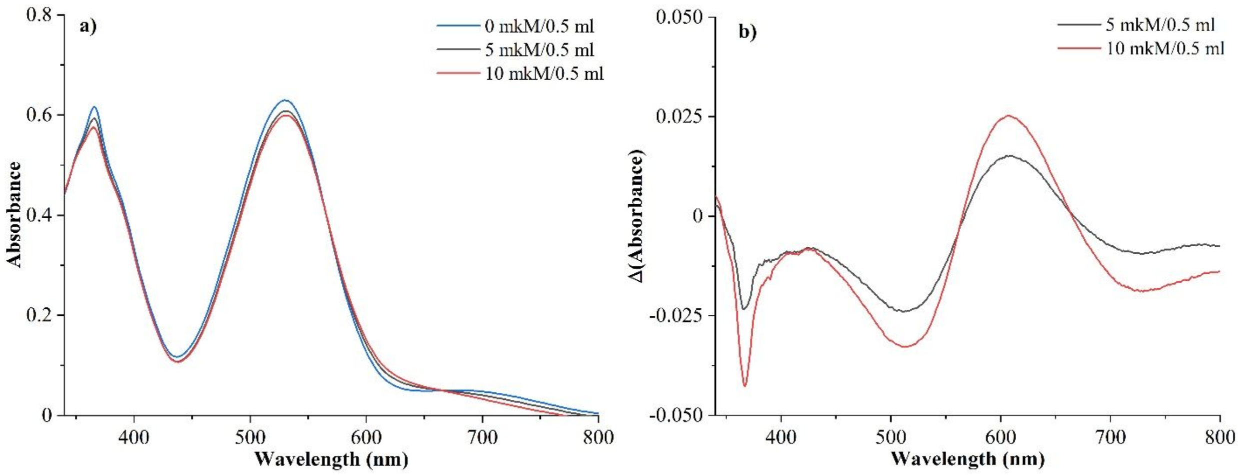

Figure S25. Absorption spectra of probe **3b** with spiked samples of serum model solution. C_{3b} = 40.0 μmol·L^{-1}, Tris-buffer (pH 7.2)/DMSO (1/5, v/v): a) raw spectra; b) obtained as $\Delta A_{\lambda} = A_{\lambda i} - A_{\lambda 0}$. Concentration of Zn^{2+} is shown in the insets.

Table S8. Recovery values of Zn^{2+} for spiked samples of serum model solution. n=3

Added, μmol·L^{-1}	Found, μmol·L^{-1}	Recovery (%)	RSD, %
5.0	5.1	102.7	0.43
10.0	10.4	104.1	0.38

Table S9. Comparison of reported colorimetric probe with proposed for detection of Zn^{2+} [6-12]

Compound	λ, nm	LOD, μmol·L^{-1}	Object (Conditions)	Interfering ions (Masking agent)	References
Dithizone (1,5-Diphenylthiocarbazone)	538	0.2	Micellar solutions (pH 9, 0.05 M diethanolamine - HCl buffer with SDS)	Co^{2+}, Pb^{2+}, Cd^{2+}	6
PAR (4-(2-Pyridylazo) resorcinol)	495	1.0	Metalloproteins (pH 7.4, 50 mM Hepes buffer with guanidine hydrochloride)	Cu^{2+}, Ni^{2+}	7
nitro-PAPS (3-[3-hydroxy-4-[(5-nitropyridin-2-yl)diazenyl]-N-propylanilino]propane-1-sulfonate)	574	0.15	Serum (pH 7.8-8.5, Tris-buffer)	Cu^{2+}, Fe^{2+} (CN^{-})	8
5-Br-PAPS (2-(5-Bromo-2-pyridiylazo)-5-[N-n-propyl-N-(3-sulfopropyl)amino]phenol)	560	15	Serum (0.5 M ammonium acetate/7.2×10^{-3} M salicylaldehyde oxime-l%(v/v) Triton X-100-0.5 M hydrochloric acid)	Fe^{2+}, Cu^{2+} (salicylaldehyde oxime, Na_3Cit)	9
TCPP (5,10,15,20-tetrakis(4-carboxylphenyl)-porphyrin)	423	0.9	Natural waters and nutritional supplement (pH 9, Pb^{2+} and 0.05 M borate buffer)	Fe^{3+} (0.1% (w/v) NH_2OH)	10
Xylenol orange (2,2′,2″,2‴-{(1,1-Dioxo-2,1λ6-benzoxathiole-3,3(1H)-diyl)bis[(6-hydroxy-5-methyl-3,1-phenylene)methylenenitrilo]}tetra acetic acid)	580	0.6	Pharmaceuticals (pH = 5-6, Acetate buffer with 1 mM CDC; stability - 3 h; with CDC up to 7 days)	Al^{3+}, Fe^{3+}, Cu^{2+}, Ni^{2+}, Pb^{2+} (NaF, EDTA)	11
Zincon (2-carboxy-2'- hydroxy-5'-sulfoformazylbenzene monosodium salt)	620	0.2	Metalloproteins (pH = 8.5 - 9.5, urea; stability - 1-2 h)	Co^{2+} in the presence of Cu^{2+}, Cu^{2+} (EDTA)	12
**3b** ((Z)-2-(benzo[d]thiazol-2-yl)-2-(5-methyl-3-oxoindolin-2-ylidene)acetonitrile)	600<br>640	0.5<br>0.4	Serum model solution (stripped of protein) (Tris-buffer (pH 7.2)/DMSO (1/5, v/v))	Co^{2+} after 30 min of prepared, Cu^{2+} (1.0 mM $Na_2S_2O_3$)	This work

References

1. Halliday, C.; Leonard, M. Selective and precise photometric titration of metal ions with EDTA using masking agents. Part III. Determination of calcium, lead and zinc in mixtures using simple inorganic ions as masks. *Analyst* **1987**, *112*, 1731-1734.
2. Smith, R.M.; Martell, A.E. Inorganic Ligands. In Critical Stability Constants; Springer, Boston, MA, 1976.
3. Rabai, G.; Epstein, I. Equilibria and kinetics of the fast interaction between copper(II) and thiosulfate ions in aqueous solution. *Inorg. Chem.* **1992**, *31*, 3239-3242.
4. Senanayake, G. Role of copper(II), carbonate and sulphite in gold leaching and thiosulphate degradation by oxygenated alkaline non-ammoniacal solutions. *Miner. Eng.* **2005**, *18*, 409-426.
5. Black, S.B. The thermodynamic chemistry of the aqueous copper-ammonia thiosulfate system. Ph.D. Thesis, Mudoch University, 2006. https://researchrepository.murdoch.edu.au/id/eprint/336/2/02Whole.pdf (accessed 2022-10-09).
6. Abdel-Latif, M. Direct determination of zinc using dithizone in micellar solution. *Anal. Lett.* **1994**, *27*, 2341-2353.
7. Säbel, C.; Shepherd, J.; Siemann, S. A direct spectrophotometric method for the simultaneous determination of zinc and cobalt in metalloproteins using 4-(2-pyridylazo)resorcinol. *Anal. Biochem.* **2009**, *391*, 74–76.
8. Makino, T. A Sensitive, direct colorimetric assay of serum zinc using nitro-PAPS and microwell plates. *Clin. Chim. Acta* **1991**, *197*, 209–220.
9. Hayashibe, Y.; Takeya, M.; Sayama, Y. Direct Determination of Zinc in Human Serum by Flow-Injection Spectrophotometric Analysis. *Anal. Sci.* **1994**, *10*, 795-799.
10. Kilian, K.; Pyrzyńska, K. Spectrophotometric study of Cd(II), Pb(II), Hg(II) and Zn(II) complexes with 5,10,15,20-tetrakis(4-carboxylphenyl)porphyrin. *Talanta* **2003**, *60*, 669–678.
11. Benamor, M.; Belhamel, K.; Draa, M. Use of xylenol orange and cetylpyridinium chloride in rapid spectrophotometric determination of zinc in pharmaceutical products. *J. Pharm. Biomed. Anal.* **2000**, *23*, 1033–1038.
12. Säbel, C.; Neureuther, J.; Siemann, S. A spectrophotometric method for the determination of zinc, copper, and cobalt ions in metalloproteins using zincon. *Anal. Biochem*. **2010**, *397*, 218–226.
